# Genetic analysis of biopsy-related droplets and its potential applications in PGT-SR patients

**DOI:** 10.3389/fendo.2026.1946046

**Published:** 2026-10-21

**Authors:** Zhiqing Huang, Yuzhi Chen, Xiaonuan Cai, Shujing Zhu, Jing Wang, Ruili Xie, Hang Shi, Aili Yu, Beihong Zheng, Yan Sun

**Affiliations:** 1 Center of Reproductive Medicine, Fujian Maternity and Child Health Hospital, Fuzhou, China; 2 Fujian Maternity and Child Health Hospital College of Clinical Medicine for Obstetrics and Gynecology and Pediatrics, Fujian Medical University, Fuzhou, China; 3 The School of Basic Medical Sciences, Fujian Medical University, Fuzhou, China; 4 The First Clinical Medical College, Fujian Medical University, Fuzhou, China; 5 Fujian Clinical Research Center for Maternal-Fetal Medicine, Fuzhou, China

**Keywords:** biopsy-related droplets, copy number variation, embryo selection, preimplantation genetic testing, repeat biopsy

## Abstract

**Purpose:**

To investigate genetic material in biopsy-related droplets (BRDs) from blastocyst biopsy and its potential applications in preimplantation genetic testing for structural rearrangements (PGT-SR).

**Methods:**

BRDs were collected from 218 blastocysts by combining three types of biopsy-related droplets (biopsy, washing, and temporary storage droplets) into ~15 µL samples. Genomic DNA amplification and copy number variation (CNV) sequencing library preparation were performed. Next-generation sequencing (NGS) was conducted on BRD libraries with concentrations ≥0.5 ng/µL.

**Results:**

69.27% of BRDs had a sequencing library concentration (CSL) ≥ 0.5 ng/µL, with a CNV detection success rate of 94.04%. CSL was significantly higher in biopsy-classified non-euploid than euploid blastocysts (P < 0.0001). Among 142 BRDs with successful CNV detection, 87.32% showed ploidy concordance with PGT findings. Complete or partial concordance was observed in 77.53% of blastocysts with structural rearrangement-related abnormalities. In 37 transferred biopsy-euploid blastocysts, clinical pregnancy rates were higher in the BRD-euploid and undetectable/non-informative BRD groups than in the BRD-non-euploid group (85.71% and 86.96% vs. 28.57%). In seven blastocysts undergoing secondary biopsy after initial testing failure, BRD and secondary biopsy results showed 85.71% ploidy concordance.

**Conclusions:**

BRD-based genetic testing may serve as an adjunct to conventional biopsy-based testing in PGT-SR.

## Introduction

Preimplantation genetic testing (PGT) combines assisted reproduction with genetic analysis, enabling chromosomal assessment of embryos via biopsy and thereby helping to reduce the risk of birth defects and adverse pregnancy outcomes by avoiding the transfer of affected embryos (1, 2). PGT is primarily classified into three categories: PGT-A (Preimplantation Genetic Testing for Aneuploidy), PGT-SR (Preimplantation Genetic Testing for Structural Rearrangements), and PGT-M (Preimplantation Genetic Testing for Monogenic Disorders) (3). Among these, PGT-SR is specifically used for couples in whom one or both partners carry chromosomal structural abnormalities, such as reciprocal translocations, Robertsonian translocations, inversions, complex rearrangements, or pathogenic microdeletions/microduplications. In carriers of balanced structural rearrangements, meiotic segregation may generate embryos with a normal or balanced chromosomal complement, as well as a high proportion of unbalanced embryos carrying chromosomal gains or losses related to the parental rearrangement. In a recent multicenter PGT-SR study of 7,750 successfully analyzed blastocysts from couples carrying balanced chromosomal rearrangements, 34.53% were classified as euploid embryos (including 16.89% carrier embryos and 17.64% non-carrier embryos), whereas 50.06% showed rearrangement-related abnormalities (including 37.32% with rearrangement-related imbalance and 12.74% with complex abnormalities involving additional *de novo* aneuploidies) (4). Transfer of an unbalanced embryo may result in implantation failure or miscarriage and, if an ongoing pregnancy is established, may lead to congenital abnormalities or developmental disorders in the offspring (5). In PGT-SR, chromosome copy-number analysis can identify such unbalanced abnormalities, whereas distinguishing chromosomally normal embryos from balanced carriers generally requires additional haplotype- or breakpoint-based analysis (4). The successful implementation of PGT technology depends on reliable biopsy techniques to obtain embryonic genetic material suitable for testing. Currently, common biopsy methods include laser-assisted techniques, mechanical methods, or a combination of both (6). Biopsy-based testing has become the mainstream approach and can achieve high analytical accuracy under validated laboratory conditions.

However, biopsy-based PGT may still be limited by embryonic mosaicism, particularly potential discordance between the trophectoderm (TE) and inner cell mass (ICM), which may prevent a small biopsy sample from fully representing the chromosomal constitution of the whole blastocyst (7, 8). In addition, it may occasionally yield inconclusive or failed results, with the rate of inconclusive blastocyst PGT diagnosis reported to be approximately 2%–6% across different testing platforms (9–13). These cases may therefore lead to the clinical dilemma of whether a repeat biopsy should be performed. Therefore, additional embryo-derived genetic material may help address these limitations of biopsy-based testing, enabling more comprehensive chromosomal assessment and serving as a potential backup source of genetic information in cases of inconclusive or failed detection. Recent studies have shown that detectable genetic material is present in spent culture medium (SCM) and blastocoelic fluid (BF) (14–16). Compared with biopsy samples derived from a localized region of the TE, embryo-derived cfDNA in these non-invasive samples has been shown to reflect chromosomal information consistent with both the TE and ICM, with reported ploidy concordance rates of 89.1% with TE biopsy and 87.0% with ICM biopsy (6, 17, 18).

Nevertheless, the mechanisms of cfDNA release remain unclear, and cfDNA in these samples is often present in low quantities and highly fragmented. These factors create inherent challenges for detection and analysis, limiting the clinical application of non-invasive testing and preventing it from replacing conventional biopsy-based testing at present (19–21). The collection of SCM requires strict protocols and may involve extended culture for optimized sampling and be affected by external interference, such as maternal contamination (7, 22), whereas BF may provide a more direct and relatively less contaminated source of embryonic genetic material but usually requires aspiration or other additional procedures, limiting its widespread clinical application (23, 24). Some studies have attempted to combine BF obtained via laser-assisted hatching with SCM to enhance cfDNA yield (22), but BF release and collection through laser-assisted hatching may be inefficient and variable among embryos (24, 25).

In PGT cycles where TE biopsy is already required, the biopsy incision, cell manipulation, and washing procedures allow the release of a considerable amount of blastocoelic fluid, and even small amounts of embryonic cellular material to enter the surrounding operational droplets. These biopsy-related droplets (BRDs), including the biopsy droplet, washing droplet, and temporary storage droplet used during blastocyst biopsy, may therefore serve as a potential and readily obtainable source of genetic material. The invasive opening during blastocyst biopsy allows substantial blastocoelic fluid to enter the surrounding droplets, making BRDs a biopsy by-product that may provide additional embryo-associated chromosomal information beyond that obtained from localized TE sampling. This may help identify additional chromosomal signals not captured by TE biopsy in embryos classified as euploid and inform repeat-biopsy decisions after inconclusive or failed testing. Our previous study demonstrated the feasibility of BRD-based genetic analysis in patients undergoing PGT-A (26). Building on these findings, the present study aimed to characterize the nucleic acid content of BRDs and explore the potential clinical utility of BRD analysis for prioritizing biopsy-euploid embryos and informing repeat-biopsy decisions in PGT-SR.

## Materials and methods

### Study participants

Patients undergoing PGT-SR at the Reproductive Medicine Center of Fujian Maternity and Child Health Hospital in 2024 were consecutively enrolled for this study. A total of 62 couples undergoing 67 PGT-SR cycles were included (218 blastocysts in total, 4 supplementary blastocysts from 3 patients in 2025 that had initial biopsy failure). After the completion of blastocyst biopsy, BRDs were collected for genetic analysis, and relevant clinical data were retrospectively collected.

### Experimental methods

#### Controlled ovarian stimulation, oocyte retrieval and sperm preparation

Controlled ovarian stimulation and oocyte retrieval were performed according to our routine protocols, as previously described (27, 28), and sperm preparation was performed using the swim-up method. Of the 67 PGT cycles, 85.07% (57/67) were conducted using the gonadotropin-releasing hormone (GnRH) antagonist protocol, while 10.45% (7/67) employed the long GnRH agonist protocol.

#### *In vitro* fertilization, embryo culture and biopsy procedure

After cumulus cell removal, MII oocytes were fertilized via intracytoplasmic sperm injection (ICSI) using a micromanipulation system (micromanipulation system: Eppendorf, Hamburg, Germany; holding and injection pipettes: Cooper, San Ramon, U.S.) with an oocyte holding pipette and an injection needle (Cooper, San Ramon, U.S.). Embryos were cultured using sequential culture methods. The sequential culture involved culturing cleavage-stage embryos (Day 1 to Day 3) in G1-PLUS medium (Vitrolife, Gothenburg, Sweden) and blastocysts (Day 4 to Day 6) in G2-PLUS medium (Vitrolife, Gothenburg, Sweden), both maintained in a tabletop incubator (COOK, Brisbane, Australia). All embryo cultures were conducted at 37 °C under conditions of 6% (V/V) CO_2_ and 5% (V/V) O_2_.

On Day 5 and Day 6 (approximately 116 and 140 hours post-fertilization, respectively), the formation of blastocysts was evaluated using the Gardner grading system (29). Blastocysts scoring 3BC, 3CB, or higher were selected for biopsy. Biopsy dishes were prepared on the day of the procedure. GM-PLUS medium (Vitrolife, Gothenburg, Sweden) equilibrated overnight was used to create biopsy droplets (BDs, 3-5 µL) and washing droplets (WDs, 3-5 µL), both covered with culture oil (Vitrolife, Gothenburg, Sweden) and incubated in the incubator (COOK, Brisbane, Australia) for over 30 minutes. Temporary storage droplets (TDs, approximately 10 µL) were prepared the day prior to the biopsy using G2-PLUS medium (Vitrolife, Gothenburg, Sweden) and equilibrated overnight under oil in the incubator (COOK, Brisbane, Australia). Biopsy was performed using a combination of laser-assisted and mechanical methods. During blastocyst expansion, a laser (laser membrane piercing device, Hamilton, USA) was used to perforate the zona pellucida, avoiding the ICM. A biopsy needle was then used to aspirate and gently separate a small number of trophectoderm (TE) cells. While withdrawing the needle, the laser was continuously applied to cut the cell junctions, ultimately obtaining 3–7 cells per sample. Each blastocyst was assigned a specific BD, WD, and TD, with the biopsy procedure typically completed within 10 minutes.

#### BRD collection, genome amplification and sequencing library construction

The procedures for sample collection and processing are detailed in **Figure 1**. Blastocysts were biopsied in the BD and subsequently washed in the corresponding WD on the biopsy dish. After the procedure, the biopsied blastocysts were placed in the TD before cryopreservation, while the biopsy cells were transferred to a commercially available sample preservation tube (PGT-XK-043, Yikon Genomics, Suzhou, China) for genetic testing. During the biopsy process, the corresponding biopsy blastocyst codes were labeled on the bottom of the biopsy dishes. After the biopsy, excess oil was removed from the biopsy and storage dishes using a pipette. For each blastocyst, the corresponding BD, WD, and TD were carefully aspirated and combined into a single sample preservation tube (approximately 15 µL of BRD). Each preservation tube was labeled with the corresponding blastocyst code.

**Figure 1:**
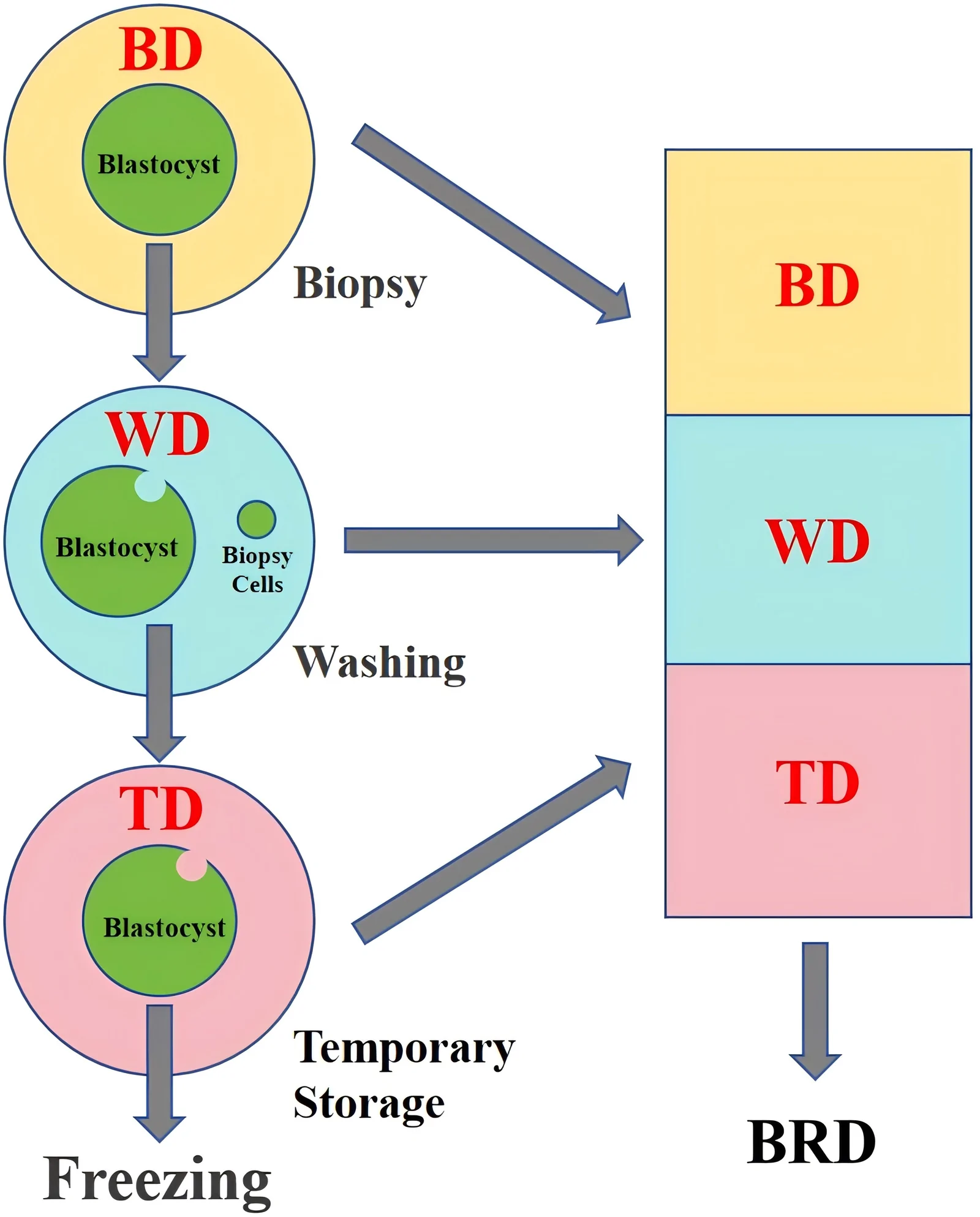
Schematic diagram of biopsy-related droplet (BRD) collection. During the blastocyst biopsy procedure, each blastocyst was sequentially exposed to three types of droplets: the biopsy droplet (BD), where the trophectoderm biopsy was performed; the washing droplet (WD), where the biopsied blastocyst and the biopsy cells were rinsed; and the temporary storage droplet (TD), where the biopsied blastocyst was placed before cryopreservation. After the procedure, the BD, WD, and TD corresponding to each blastocyst were fully aspirated and combined into a single sample tube, yielding 10-15 µL of biopsy-related droplet (BRD) for subsequent genomic analysis.

Genomic DNA from biopsy cells and their associated BRDs was amplified using the ChromInst kit (YIKON Genomics, Suzhou, China), according to the manufacturer’s instructions. Genome amplification was performed using the multiple annealing and looping-based amplification cycles (MALBAC) method, followed by copy number variation (CNV) library construction. CNV libraries were purified using CMPure magnetic beads provided in the ChromInst kit following the recommended protocol, and the concentrations of the purified libraries were measured using a Qubit 3.0 (Thermo Fisher, Waltham, USA). Blank droplet mixtures of equal volume, which were not derived from any patient sample, were used as negative controls to assess background nucleic acids from reagents, while commercially available trisomy 21 fibroblast cells (XK-007, YIKON Genomics, Suzhou, China) were used as positive controls.

#### Next-generation sequencing and CNV analysis

CNV libraries from biopsy cells with concentrations ≥ 5.0 ng/µL, and those from BRDs with concentrations ≥ 0.5 ng/µL, were sent to YIKON Genomics (Suzhou, China) and Decode Biomedical Technology Co., Ltd. (Shanghai, China), respectively, for sequencing. The sequencing was performed using the DNBSEQ-T7 platform (BGI, Shenzhen, China) in PE150 paired-end sequencing mode, yielding a minimum of 2.0 million reads per sample. CNV analysis was conducted using ChromGo software (version V1.9.1, YIKON Genomics, Suzhou, China) following standard procedures. Reported abnormalities included chromosomal aneuploidy, fragment deletion/duplication of ≥ 4 Mb for TE and ≥ 10 Mb for BRD (a lower resolution threshold was applied to BRD samples because of the lower quantity and more fragmented nature of BRD-derived DNA), and mosaic abnormalities of 10 Mb and above with a proportion of ≥ 30%. Mosaicism was defined as a proportion of abnormal segments between 30% and 70%, euploidy was defined as a proportion of abnormal segments < 30%, and aneuploidy was defined as a proportion of abnormal segments > 70%. Non-euploidy included mosaic or aneuploidy. The reference genome used for CNV analysis was GRCh37/hg19. BRD samples with a CSL <0.5 ng/µL were considered insufficient for NGS and were classified as undetectable. For samples with a CSL ≥0.5 ng/µL, those that underwent sequencing but failed to generate a CNV profile of sufficient quality for reliable interpretation were reported as non-informative (30–32). In this study, non-informative results referred to samples with insufficient analytical quality for CNV interpretation, such as poor signal quality or amplification performance, rather than samples showing complex or chaotic chromosomal profiles. These samples were excluded from CNV concordance analyses between BRDs and biopsy cells requiring interpretable BRD results but were retained in descriptive and clinical outcome analyses.

#### Statistical analysis

Data analysis was performed using SPSS 26.0 statistical software. For quantitative data with normal distribution and homogeneity of variance, results were reported as mean ± standard deviation (x̄ ± s), and compared using independent samples *t*-tests. Categorical data were presented as count and percentage (%), and differences between groups were assessed using the Chi-square (χ^2^) test or Fisher’s exact test. A P-value < 0.05 was considered statistically significant, and a P-value < 0.01 was considered highly significant.

#### Ethics statement

This study was approved by the Ethics Committee of Fujian Maternity and Child Health Hospital (approval No. 2024KY135). Written informed consent was obtained from all participants. The study was conducted in accordance with the ethical standards of the responsible committee on human experimentation and with the Declaration of Helsinki and its later amendments.

## Results

### CSL values and CNV detection results of BRD samples

Among the 62 included couples (67 PGT cycles), 42 cases involved balanced reciprocal translocations (45 PGT cycles, 136 blastocysts), 13 cases involved Robertsonian translocations (14 PGT cycles, 58 blastocysts), and 7 cases presented with other types of chromosomal structural abnormalities, including inversions, microdeletions, microduplications, and complex rearrangements (8 PGT cycles, 24 blastocysts). In total, 218 blastocysts were available for biopsy. Of these, 211 biopsy cell samples yielded valid CNV results, while 7 failed to yield valid results initially but were successfully analyzed following a second biopsy. All 218 BRD samples underwent whole-genome amplification and CNV library construction. The mean CSL of the three negative controls was 0.246 ng/µL. Among the 218 BRD samples, 151 (69.27%) reached the predefined sequencing threshold of CSL ≥0.5 ng/µL and proceeded to NGS, whereas the remaining 67 (30.73%) had insufficient CSL and were not sequenced. Among the 151 sequenced samples, 118 had a CSL ≥1.0 ng/µL, while 33 had CSL values between 0.5 and 1.0 ng/µL. Interpretable CNV results were obtained in 115/118 (97.46%) and 27/33 (81.82%) samples, respectively. Overall, 142 of the 151 sequenced BRD samples yielded interpretable CNV results, corresponding to a CNV detection success rate of 94.04%. When all 218 collected BRD samples were considered, the overall informative yield was 65.14% (142/218). All negative controls yielded no interpretable CNV results, whereas the positive controls showed trisomy 21 (+21) as expected.

### Analysis of factors influencing nucleic acid content in BRD samples

A threshold of 1.0 ng/µL was used to categorize blastocysts into the high CSL group (HCSL, ≥ 1.0 ng/µL, n = 118) and the low CSL group (LCSL, < 1.0 ng/µL, n = 100). Comparisons were conducted between the two groups with respect to several parameters, including the ages of both female and male participants, the number of retrieved and mature oocytes, the quantity of high-quality Day 3 (D3) embryos, and the total and high-quality blastocyst counts. Additionally, we examined BMI of the female partner, female infertility type and duration, semen quality, the developmental day of the blastocyst (D5 or D6), blastocyst morphological grade, ICM and TE grade, the biopsy operator, CNV result of biopsy cells, the sex of the carrier of chromosomal structural abnormalities, and the number of biopsy cells. The results are presented in **Table 1**, including the sample sizes, proportions, and BRD CSL values for morphological grade, TE grade, and ploidy status. CSL values of the BRDs were significantly associated with blastocyst morphological grade (P = 0.007), TE grade (P = 0.005), and chromosomal ploidy determined by biopsy (P < 0.0001). Compared with blastocysts with higher morphological grades and TE grades, blastocysts with lower morphological grades and TE grades had higher CSL values in their BRDs. Additionally, BRDs of biopsy-classified non-euploid blastocysts exhibited significantly higher CSL levels than those of biopsy-classified euploid blastocysts.

**Table 1:** Factors associated with BRD CSL and CSL distribution by blastocyst characteristics (N = 218).

A. Comparisons between the LCSL and HCSL groups			
Factors	Group values, mean ± SD or n	Test statistic	P value
Female Age (Years)	LCSL: 30.49 ± 3.49; HCSL: 31.46 ± 3.83	1.936	0.054
Male Age (Years)	LCSL: 33.11 ± 4.56; HCSL: 33.81 ± 4.78	1.106	0.270
Number of Retrieved Oocytes	LCSL: 16.69 ± 6.93; HCSL: 17.13 ± 7.55	0.442	0.659
Number of MII Oocytes	LCSL: 13.58 ± 5.88; HCSL: 13.79 ± 6.07	0.256	0.798
Number of High-Quality Day 3 Embryos	LCSL: 8.87 ± 3.54; HCSL: 9.01 ± 3.70	0.281	0.779
Number of Blastocysts Formed	LCSL: 7.71 ± 3.11; HCSL: 7.68 ± 3.26	0.074	0.941
Number of High-Quality Blastocysts	LCSL: 4.80 ± 2.48; HCSL: 4.39 ± 2.56	1.196	0.233
Body Mass Index	Normal: 129; Abnormal: 89	0.052	0.819
Type of Female Infertility	Primary: 45; Secondary: 173	2.430	0.119
Duration of Female Infertility	<2 years: 152; ≥2 years: 66	2.435	0.119
Semen Quality	Normal: 137; Abnormal: 81	2.998	0.083
Blastocyst Developmental Day	Day 5: 65; Day 6: 153	2.372	0.124
Blastocyst Morphological Grade	Good: 26; Fair: 119; Poor: 73	10.029	0.007
ICM Grade	A: 8; B: 191; C: 19	2.617	0.270
TE Grade	A: 12; B: 141; C: 65	10.481	0.005
Biopsy Operator	Operator 1: 34; Operator 2: 39; Operator 3: 53; Operator 4: 92	2.079	0.556
CNV Result of Biopsy Cells	Euploid: 98; Non-euploid: 120	30.001	<0.0001
Sex of the Structural Rearrangement Carrier	Female: 94; Male: 124	1.134	0.287
Number of Biopsy Cells	<5: 82; ≥5: 136	0.538	0.463
B. BRD CSL by Blastocyst Morphological Grade, TE Grade, and Biopsy-Based Ploidy			
**Blastocyst characteristics**	**Category**	**n (%)**	**BRD CSL (ng/µL), mean ± SD**
Blastocyst Morphological Grade	Good	26 (11.93)	1.97 ± 2.31
Blastocyst Morphological Grade	Fair	119 (54.59)	2.49 ± 4.22
Blastocyst Morphological Grade	Poor	73 (33.49)	3.00 ± 4.35
TE Grade	A	12 (5.50)	1.59 ± 1.51
TE Grade	B	141 (64.68)	2.42 ± 3.99
TE Grade	C	65 (29.82)	3.16 ± 4.55
CNV Result of Biopsy Cells	Euploid	98 (44.95)	1.51 ± 2.27
CNV Result of Biopsy Cells	Non-euploid	120 (55.05)	3.49 ± 4.95

BRD, biopsy-related droplets; BMI, body mass index; CNV, copy number variation; CSL, concentration of the sequencing library; ICM, inner cell mass; MII, metaphase II; SD, standard deviation; TE, trophectoderm. LCSL denotes CSL <1.0 ng/µL (n = 100), and HCSL denotes CSL ≥1.0 ng/µL (n = 118). In Panel B, percentages are calculated using all 218 blastocysts as the denominator and may not sum to 100% because of rounding. Ploidy was determined from TE biopsy results. Test statistics and P values in Panel A refer to the original LCSL/HCSL group analyses; CSL values in Panel B are descriptive summaries and were not separately tested in this table. BMI was classified as normal (18.5 ≤ BMI <24.0 kg/m^2^) or abnormal (BMI <18.5 or ≥24.0 kg/m^2^). Abnormal semen quality refers to the diagnosis of at least one of the following: oligozoospermia, asthenozoospermia, or teratozoospermia. High-quality Day 3 embryos were defined as cleavage-stage embryos with a score of II/5 or above, based on the Istanbul Consensus. High-quality blastocysts were defined as blastocysts with a morphological grade of 4BB or higher, according to the Gardner grading system. Blastocyst morphological grade, ICM grade, and TE grade were assessed according to the Gardner grading system. Good morphological grade indicates that both ICM and TE were graded B or above, with at least one graded A; fair grade indicates that both were graded B; poor grade indicates that either ICM or TE was graded C. Biopsy cell count was categorized as <5 or ≥5 cells.

### Comparison of CNV results of biopsy cells and their corresponding BRDs

Among the 218 BRD samples, 76 (34.86%) did not yield interpretable CNV results, including 67 samples with CSL <0.5 ng/µL that were not subjected to NGS and 9 samples that yielded non-informative results after sequencing. Because no interpretable BRD CNV result was available for these samples, concordance with the corresponding biopsy-cell results could not be evaluated. Therefore, concordance analysis was performed on the remaining 142 BRDs with interpretable CNV results and their corresponding biopsy-cell results. Considering the expected fragmented nature of BRD-derived DNA, the comparison focused primarily on major chromosomal abnormalities shared between BRD and biopsy results, while the sizes of abnormal segments and the mosaicism ratios were considered auxiliary parameters. However, for abnormalities on the same chromosome, if one sample exhibits a gain and the other a deletion, the results would be considered inconsistent. The findings were classified into three categories (**Table 2**, **Figure 2**, **Supplementary Table 1**).

**Table 2:** CNV result consistency between biopsy cells and corresponding BRDs (6 representative samples).

Blastocyst code	CNV of biopsy cell	CSL (ng/µL)	CNV of BRD	Result consistency
CC1	Euploid	4.920	Euploid	Completely Consistent
CC2	del(2)(q35q37.3)(~22.20Mb), dup(16)(q13q24.3)(~33.15Mb)	5.800	del(2)(q35q37.3)(~22.20Mb), dup(16)(q13q24.3)(~33.35Mb,~69%)	
PC1	-21,-22	3.840	+1(~47%),-21,-22(~57%)	Partially Consistent
PC2	del(10)(p15.3p15.2)(~3.80Mb), dup(17)(p13.3p13.1)(~10.40Mb)	3.640	dup(17)(p13.3p12)(~11.00Mb)	
CI1	Euploid	0.560	-14(~43%)	Completely Inconsistent
CI2	dup(10)(q11.21q26.3)(~92.53Mb,~33%)	3.240	del(7)(q22.1q36.3)(~55.64Mb,~41%)	

BRD, Biopsy-related droplets; CNV, Copy number variation; CSL, Concentration of the sequencing library; Completely Consistent: Both TE and corresponding BRD samples were either euploid or exhibited identical CNV findings without additional discrepancies; Partially Consistent: TE and BRD samples shared at least one identical CNV, but additional non-overlapping CNVs were present in one or both samples; Completely Inconsistent: TE and BRD samples showed entirely different chromosomal findings with no overlapping CNVs.

**Figure 2:**
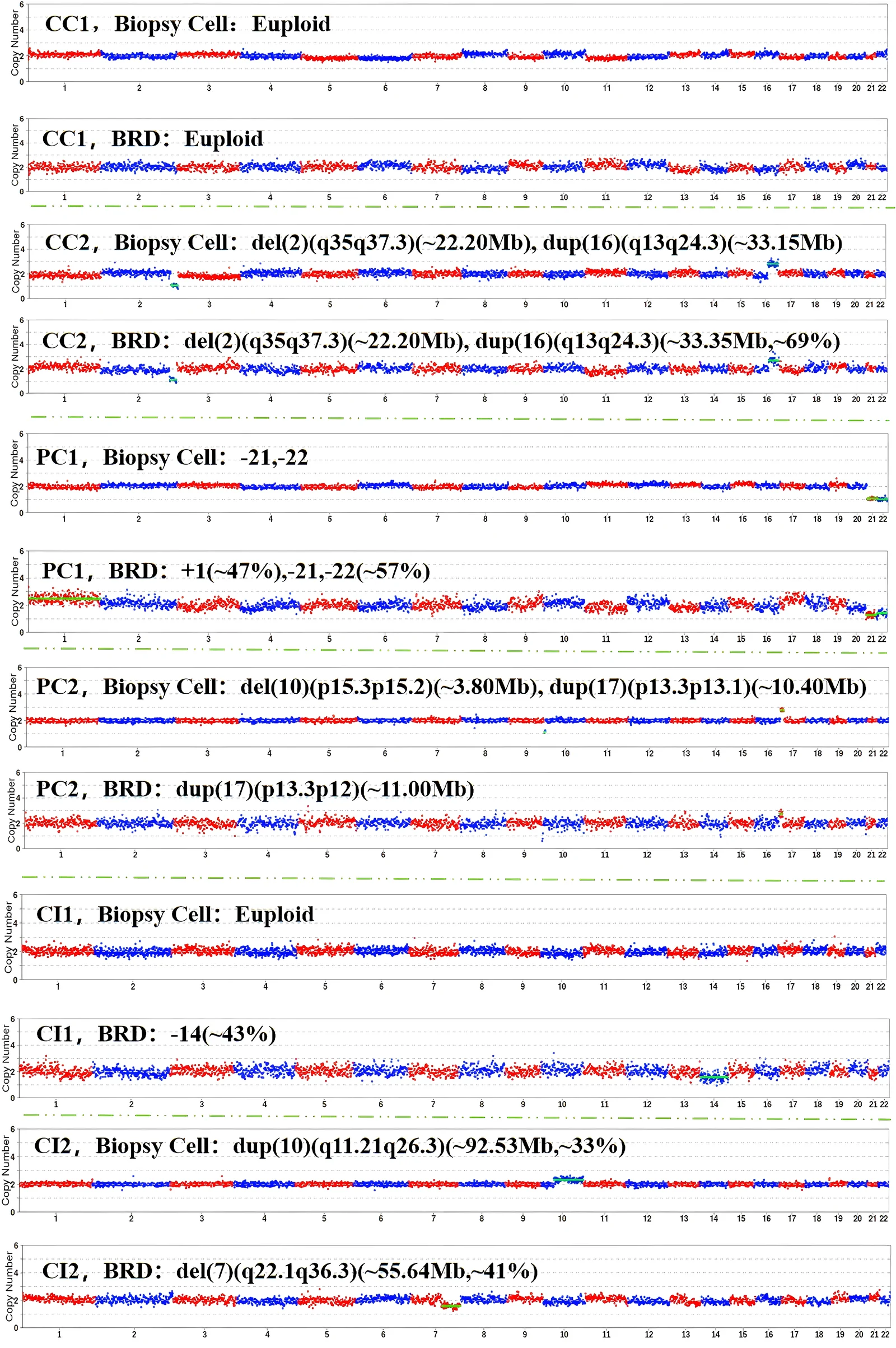
CNV profiles of biopsy cells and corresponding BRDs (6 representative samples).

Category I (complete concordance): 40.85% (58/142) of the cases exhibited identical CNV results between BRD and biopsy samples. This included instances where both samples were euploid (e.g., CC1) or displayed generally the same CNV profile (e.g., CC2).

Category II (partial concordance): 38.73% (55/142) of the cases showed overlapping CNV profiles between BRD and biopsy samples, with some different CNVs (e.g., PC1 and PC2).

Category III (complete discordance): 20.42% (29/142) of the cases showed entirely different CNV results between BRD and biopsy cells. This included instances where one sample was euploid while the other was non-euploid (e.g., CI1), as well as those with non-overlapping CNVs (e.g., CI2).

From the perspective of chromosomal ploidy, discordance between biopsy cells and BRDs was defined as one being euploid and the other non-euploid (18 samples, e.g., CI1); otherwise, the results were considered concordant. Overall, among the 142 samples with interpretable CNV results (**Supplementary Table 1**), there was a relatively high consistency in chromosomal ploidy between BRDs and their corresponding biopsy cells, reaching 87.32% (124/142). These 124 ploidy-concordant cases comprised all 113 Category I and II cases and 11 Category III cases. Overall, these findings underscore the potential reliability of BRD-based testing as a complementary approach for assessing chromosomal abnormalities.

For blastocysts from patients with chromosomal structural abnormalities, abnormal findings related to structural rearrangement-related chromosomes identified by conventional PGT are generally considered reliable. Therefore, such samples provide a reliable basis for evaluating the accuracy and reliability of BRD-based detection. Among the 120 blastocysts classified as non-euploid based on PGT results included in this study, 74.17% (89/120) were found to carry abnormalities related to chromosomal structural rearrangements. Focusing on the 89 blastocysts with structural rearrangement-related abnormalities detected by biopsy-based PGT-SR, complete or partial concordance between BRD and biopsy results was observed in 77.53% (69/89) of cases, while ploidy concordance was observed in 83.15% (74/89). Notably, BRDs identified abnormalities involving the same chromosome(s) affected by the structural rearrangement as the TE biopsy in 79.78% (71/89) of cases. The remaining 18 blastocysts (20.22%, 18/89) showed no corresponding abnormalities in their BRD samples, including 9 with CSL values too low for detection, 3 with non-informative BRD results, and 6 with abnormalities unrelated to the chromosomal structural rearrangements. Among the 6 blastocysts showing complete discordance between BRD and PGT results, only 1 showed discordance at the euploid/non-euploid classification level, with the BRD result classified as euploid.

### Association of euploid blastocyst transfer outcomes and BRD detection results

Among the 218 embryos, 98 were identified as euploid by TE biopsy, of which 37 were transferred (15 ongoing pregnancies, 12 live births, 1 miscarriage, 2 biochemical pregnancies, and 7 non-pregnancies). As of April 30, 2026, all 15 ongoing pregnancies had progressed beyond 12 weeks of gestation. All 12 live-birth cases underwent prenatal diagnosis, and chromosomal microarray analysis (CMA; resolution, 100 kb) of amniotic fluid samples showed no significant chromosomal abnormalities in the fetuses. Among these 37 transferred TE-euploid blastocysts, 7 had euploid BRD results, 7 had non-euploid BRD results, and 23 had undetectable or non-informative BRD results, including 2 non-informative cases after sequencing and 21 cases with insufficient CSL (<0.5 ng/µL) that did not undergo NGS (**Table 3**).

**Table 3:** Association between blastocyst transfer outcomes and BRD CNV results.

BRD CNV	Total	No pregnancy	Ongoing pregnancy	Biochemical pregnancy	Miscarriage	Live birth	Clinical pregnancy
Undetectable/non-informative	23	2	13	1	0	7	20
Euploid	7	1	1	0	0	5	6
Non-euploid — group subtotal	7	4	1	1	1	0	2
-3 (~63%)	1	0	0	0	1	0	1
-9 (~52%)	1	0	0	1	0	0	0
-3 (~52%), dup(5)(p14.3q35.3) (~160.42 Mb, ~34%), dup(20)(p13q13.12) (~44.00 Mb, ~35%)	1	0	1	0	0	0	1
-5 (~40%), +17 (~54%), dup(20)(q11.21q13.33) (~33.53 Mb)	1	1	0	0	0	0	0
dup(4)(q12q35.2) (~136.65 Mb, ~56%), dup(13)(q12.13q13.3) (~12.00 Mb)	1	1	0	0	0	0	0
-2 (~31%), dup(3)(p26.3p24.3) (~23.40 Mb), del(4)(q21.21q35.2) (~109.40 Mb), -10 (~32%)	1	1	0	0	0	0	0
+15, +20 (~59%)	1	1	0	0	0	0	0
Overall	37	7	15	2	1	12	28

RD, biopsy-related droplets; CNV, copy number variation; CSL, concentration of the sequencing library; NGS, next-generation sequencing. The undetectable/non-informative group includes 21 cases with insufficient CSL (<0.5 ng/µL) that did not undergo NGS and 2 cases with non-informative results after sequencing. Clinical pregnancy includes ongoing pregnancy, miscarriage, and live birth.

Among transferred blastocysts with informative BRD results, clinical pregnancy occurred in 85.71% (6/7) of those with euploid BRD results (5 live births, 1 ongoing pregnancy, and 1 non-pregnancy) and in 28.57% (2/7) of those with non-euploid BRD results (1 ongoing pregnancy, 1 miscarriage, 1 biochemical pregnancy, and 4 non-pregnancies). The remaining 23 transferred blastocysts had undetectable or non-informative BRD results (21 with CSL <0.5 ng/µL and 2 non-informative after sequencing), of which 20 achieved clinical pregnancy, yielding a clinical pregnancy rate of 86.96% (20/23).

Thus, the BRD-euploid group showed a markedly higher clinical pregnancy rate than the BRD-non-euploid group (85.71% vs. 28.57%), while the undetectable/non-informative group also showed a similarly favorable rate of 86.96% (20/23).

### Analysis of BRD samples from blastocysts with failed initial biopsy detection

7 blastocysts that had no valid results from the initial biopsy were thawed using a commercial solution (Kitazato Corporation, Fuji, Japan) according to the manufacturer’s protocol. After blastocoel re-expansion, a second biopsy was performed. The procedures for the second biopsy were identical to those used in the initial biopsy. CNV detection was conducted on both the second biopsy cells and the BRDs from both biopsies.

All BRD samples collected from the second biopsy exhibited low CSL levels (<0.5 ng/µL), making NGS unfeasible. In contrast, the BRD samples from the initial biopsy included 1 (NA1) with an ultra-low CSL (0.324 ng/µL) that could not undergo NGS (**Table 4**), and 6 with CSLs ≥ 0.5 ng/µL that yielded successful CNV detection (CC8, CC4, PC6, PC53, PC54, and PC55). Among these, 1 was euploid and completely matched the second biopsy result. The other 5 BRDs were non-euploid: 1 showed full concordance with the second biopsy results; and 4 showed partial concordance with the second biopsy results, maintaining consistent chromosomal ploidy. Overall, among the 7 blastocysts with failed initial biopsy cell detection, the BRD samples from the initial biopsy and the second biopsy cells showed concordant euploid/non-euploid classification in 85.71% (6/7) of cases, with mosaic and aneuploid results both classified as non-euploid.

**Table 4:** CNV consistency between second biopsy cells and corresponding first BRDs.

Blastocyst code	Blastocyst developmental day	Blastocyst morphological grade	Second biopsy cell CNV	BRD CNV	Consistency
NA1	D5	4AA	Euploid	Unable to Detect	—
CC8	D6	4BC	Euploid	Euploid	complete concordance
CC4	D6	4BB	-X,-16	-X(~56%),del(16)(q12.1q24.3)(~41.35Mb,~38%)	
PC6	D5	4BB	dup(2)(q32.2q37.3)(~52.00Mb),del(9)(p24.3p24.1)(~4.80Mb)	dup(2)(q32.3q37.3)(~46.70Mb,~54%)	partial concordance
PC53	D6	4BB	+14	del(2)(q32.2q36.3)(~39.00Mb),del(11)(q22.1q23.3)(~19.50Mb,~70%),del(13)(q33.1q34)(~11.67Mb),+14,del(18)(q21.31q23)(~17.50Mb)	
PC54	D5	4BB	-16	-X(~52%),+2p(~33%),-2q(~45%),+3,+6p(~66%),+6q,+11q,+14(~55%),-16(~61%)	
PC55	D6	4BC	+13	del(1)(p36.33p36.21)(~15.00Mb),+13	

BRD, Biopsy-related droplets; CNV, Copy number variation; D5 indicates Day 5; D6 indicates Day 6. “—” indicates data not applicable.

## Discussion

### Application basis of genetic testing on BRDs

PGT currently relies primarily on invasive biopsy procedures, which also generate biopsy-related droplets (BRDs) as byproducts. However, whether these droplets contain genetic material and whether they have potential clinical applications remain unclear. In this study, we collected BRDs for genetic analysis to explore their characteristics and evaluate their potential as an auxiliary tool for more comprehensive and precise assessment of embryonic chromosomal status, repeat-biopsy decision-making, and other specific clinical scenarios. Biopsy is an invasive procedure that releases a large amount of blastocoelic fluid (BF) and may introduce other embryonic cellular material into BRDs, making these droplets a potential source of genetic material for testing. In this study, 69.27% of BRD samples had CSL values ≥ 0.5 ng/μL and showed a high CNV detection success rate of 94.04%, while among these samples, 78.15% had CSL values ≥ 1.0 ng/μL, with a higher CNV detection success rate of 97.46%, supporting the presence of detectable genetic material in BRDs and the feasibility of BRD-based chromosomal CNV analysis.

In this study, undetectable or non-informative BRD CNV results were more common in TE-euploid than TE-non-euploid blastocysts (57.14% [56/98] vs. 16.67% [20/120]), suggesting that such results should not be interpreted simply as unfavorable findings. For non-invasive or biopsy-related samples with insufficient cfDNA or failed detection, such results may be related to lower amounts of amplifiable embryo-derived DNA in samples from some euploid or higher-quality blastocysts (15, 30), as well as poor amplification performance associated with the low-input and fragmented nature of embryo-derived DNA (31, 32). Multiple studies have shown that embryos corresponding to such samples can still achieve favorable implantation and pregnancy rates, similar to those observed in embryos whose non-invasive samples are tested euploid (33, 34). Therefore, undetectable or non-informative BRD results should not be interpreted simply as adverse or meaningless findings, since they may still have value in auxiliary embryo evaluation.

Additionally, BRDs from non-euploid blastocysts exhibited significantly higher CSL values than those from euploid blastocysts. This may reflect differences in the amount or composition of nucleic acids released or introduced during biopsy among embryos with different chromosomal ploidy status, although the biological origin of embryo-associated cfDNA remains incompletely understood and is thought to involve multiple factors, including apoptosis and normal cellular metabolism (24, 25). In terms of morphology, BRD CSL values were also significantly associated with blastocyst morphological grade and TE grade, whereas no significant association was observed with developmental day. This finding appears to differ from a previous report showing that blastocyst morphological grade did not significantly affect cfDNA levels in non-invasive samples (24). These findings may suggest that poorer-quality or non-euploid blastocysts may be associated with increased cellular stress, apoptosis, or fragmentation, which may promote the release of embryo-derived nucleic acids and their introduction into BRDs during biopsy, thereby providing more detectable genetic material for CNV analysis and contributing to higher CSL values.

### Concordance of BRD analysis with PGT-SR results

Prior studies have shown that the ploidy concordance rate between non-invasive testing and TE biopsy-based testing ranges from 30% to 100% (15, 34, 35). A previous SCM/BF-based non-invasive chromosomal screening (NICS) study reported a ploidy concordance of 78.1% in the chromosomal rearrangement group after excluding contaminated or failed samples (17). In this study, interpretable CNV results, 79.58% (113/142) showed complete or partial concordance with the corresponding biopsy results, and the chromosomal ploidy concordance rate was 87.32% (124/142), with an overall informative yield of 65.14% (142/218), noting that different CNV resolution thresholds were applied to TE and BRD samples. When focusing on blastocysts with structural rearrangement-related abnormalities detected by biopsy-based PGT-SR, complete or partial concordance was observed in 77.53% (69/89) of cases, and the ploidy concordance rate reached 83.15% (74/89). However, in this subgroup, corresponding abnormalities were still not detected in 20.22% (18/89) of cases. These findings suggest that BRD-based analysis may provide supplementary information in the PGT-SR setting, but its limitations should be carefully considered.

Among the 18 samples without corresponding structural rearrangement-related abnormalities in BRDs, 9 had undetectable BRD CNV results, and 3 yielded non-informative results. These undetectable or non-informative results may be associated with limited amounts of amplifiable embryo-derived DNA and poor amplification performance related to the low-input and fragmented nature of these samples (31, 32). Previous studies have shown that non-invasive embryo-related samples may contain non-embryonic DNA in addition to embryo-derived DNA, which can interfere with the interpretation of informative genetic signals (36, 37). Moreover, highly fragmented DNA may increase amplification noise and reduce CNV profile stability (38). Notably, 5 of the 9 samples with undetectable BRD CNV results were collected more than 1 day after biopsy, suggesting a possible effect of collection timing.

In the remaining 6 cases, abnormalities were detected in BRDs, but the BRD results were discordant with the PGT-SR findings. This discordance may be related to both the complex nature of BRD samples and the technical difficulty of detecting structural rearrangement-related abnormalities. Previous studies have shown that non-invasive embryo-associated samples may contain highly fragmented DNA and DNA from non-embryonic sources, which can interfere with CNV profile stability and the interpretation of embryo-derived genetic signals, potentially contributing to discordant CNV results (36, 37). In addition, previous PGT-SR studies have suggested that accurate detection of structural rearrangement-related abnormalities may require more precise breakpoint mapping or linkage analysis, whereas conventional chromosome screening methods still have limitations in complex regions (39). Therefore, weak or unstable local signals in BRD samples may lead to missed or discordant detection of corresponding structural rearrangement-related abnormalities.

### Application in assisting the selection of euploid blastocysts

Recent studies have revealed that the occurrence of mosaicism in embryos may be more prevalent than previously estimated (40, 41). However, TE-biopsy euploid results may still be affected by sampling limitations, particularly in the presence of mosaicism (21, 42). In this study, among the 37 transferred TE-euploid blastocysts, the clinical pregnancy rate was markedly higher in the BRD-euploid group than in the BRD-non-euploid group (85.71% vs. 28.57%), consistent with a similar pattern reported in a previous NICS study (71.2% vs. 54.5%) (43). Notably, 20/23 (86.96%) blastocysts with undetectable or non-informative BRD results achieved clinical pregnancy. Similarly, Magli et al. reported a higher clinical pregnancy rate after failed than successful BF amplification (77% vs. 37%) (15), suggesting that failure to obtain an interpretable BRD result may not necessarily indicate an unfavorable reproductive outcome. Therefore, these findings suggest that BRD-based genetic analysis may provide supplementary information for the assessment of blastocysts classified as euploid by TE biopsy, consistent with observations from previous NICS studies.

### Application in assisting decision-making for a second biopsy

Currently, there is a lack of simple and effective auxiliary methods to support decision-making regarding repeat biopsy, particularly in assessing the potential benefits of such repeat procedures from the technical perspective. Non-invasive samples have been suggested as backup options in cases of testing failures (21). However, due to the low incidence of detection failures, these studies do not provide concrete evidence to support their clinical use. In PGT, inconclusive results or test failures may still occur, with the rate of inconclusive blastocyst PGT diagnosis reported to be approximately 2%–6% across different platforms, including NGS, SNP arrays, and array CGH, primarily due to poor sample quality, biopsy-related procedural factors, and limitations in genetic testing methodologies (9–13). When these failures occur, a second biopsy may be considered to obtain a definitive diagnosis. However, a second biopsy necessitates thawing, re-biopsy, and re-freezing of blastocysts, which can adversely affect the developmental potential of the embryos (44). Additionally, the high costs and complexity associated with PGT make repeat biopsies a significant financial and procedural burden on patients. On the other hand, avoiding a second biopsy may lead to discarding embryos with potential for successful pregnancy. Thus, patients and clinicians face a trade-off when deciding whether to proceed with a second biopsy.

In this study, 5 BRDs from initial biopsies detected non-euploidy, and the results from second biopsies of corresponding blastocysts also confirmed non-euploidy, suggesting that BRD findings may provide supplementary information when considering the need for repeat biopsy. In contrast, 2 BRDs from initial biopsies either failed to detect or confirmed euploidy, and the results from second biopsies of corresponding blastocysts also showed euploidy, suggesting no discordant non-euploid findings at the ploidy level. Furthermore, the testing results from BRD samples obtained during the first biopsy of 2 blastocysts were completely consistent with their second biopsy results (**Table 4**). This suggests that the initial failures might result from incomplete transfer of cells during the first biopsy. Therefore, BRD-based genetic analysis may provide supplementary information when repeat biopsy is being considered. These findings support the potential value of BRD analysis as an additional source of information in the evaluation of cases with an initial biopsy-testing failure.

### Potential application in mosaic embryos and investigation of failed euploid embryo transfer

Mosaic embryo transfer decision-making remains challenging, even though the transfer of selected mosaic embryos has become increasingly considered in clinical practice (45–47). Because TE biopsy provides only a localized chromosomal profile, it cannot fully determine the actual origin or proportion of mosaicism, particularly its distribution between the TE and ICM (48, 49). In this context, BRD-based analysis may provide supplementary information by offering an additional chromosomal signal that may not be captured by biopsy-based PGT, which may provide additional context when evaluating embryos with possible mosaicism. However, its results should be interpreted with caution, as the true chromosomal constitution of mosaic embryos may not be fully reflected by a single biopsy or non-invasive sample and may also be influenced by sampling location, cfDNA quantity, technical variability, and the uncertain relative contributions of different DNA sources within BRDs.

When implantation failure occurs after the transfer of embryos classified as euploid by PGT, identifying the underlying cause is clinically important, as both embryonic and non-embryonic factors may be involved (50). In this context, stored BRD samples could be thawed and tested for CNVs as a retrospective adjunct to investigate possible contributors. A non-euploid BRD result may raise the possibility of an embryonic contribution to transfer failure, such as undetected mosaicism or biopsy-sampling limitations. Conversely, if the BRD result shows no chromosomal abnormality (e.g. euploid), especially in patients with repeated transfer failures despite consistently normal BRD findings, non-embryonic factors, including impaired endometrial receptivity, uterine pathology, or other maternal conditions, should be further considered, may also warrant consideration (51, 52). However, because the number of transferred euploid blastocysts in this study was limited, further studies with larger sample sizes are needed to validate this potential application.

### General principles and limitations of BRD-based genetic testing

Routine BRD testing for all blastocysts is not advocated. Instead, relevant BRD samples may be thawed and tested as needed to assist embryo evaluation and clinical decision-making. Due to the low quantity and highly fragmented nature of cfDNA in BRD and SCM samples, as well as the possible presence of genetic material from uncertain or non-embryonic sources, the application of BRD-based testing or NICS in clinical diagnostics, particularly those requiring high precision, remains premature (25). However, when clear chromosomal abnormalities are detected in BRD samples, these findings may provide useful information for blastocyst prioritization and help identify blastocysts with potentially reduced reproductive potential. Indeed, some studies have suggested that even cfDNA concentration alone may help guide the optimal transfer sequence of fresh blastocysts (30).

Based on our findings, a proposed hypothesis-generating framework for the potential interpretation of BRD results is summarized in **Table 5**. In cases of initial PGT failure, if the BRD result may provide supplementary information when considering whether repeat biopsy is warranted. For blastocysts classified as euploid by biopsy-based PGT-SR, euploid or undetectable BRD results may provide supportive information for embryo prioritization, whereas non-euploid BRD results may provide less supportive information when considering embryo prioritization. The same principles may be cautiously applied to low-level mosaic blastocysts, defined in this context as those with <50% mosaicism based on TE biopsy. High-level mosaic blastocysts (≥50% mosaicism) are generally not considered for transfer in our clinical practice. For patients with repeated failed transfers of euploid embryos, if the BRD result shows no chromosomal abnormality, non-embryonic contributors may warrant further consideration. Conversely, a non-euploid BRD result may suggest the presence of adverse embryo-related chromosomal factors, including possible chromosomal discordance not fully captured by localized TE biopsy.

**Table 5:** Hypothesis-generating framework for BRD-guided clinical interpretation in PGT-SR.

CNV-seq result of biopsy cells/transfer outcome	CNV-seq result of thawed corresponding biopsy-related droplet (BRD)	Potential clinical interpretation
Detection failure or suspected abnormality	Euploid or undetectable	May support consideration of secondary biopsy
	Non-euploid	May provide less supportive information for secondary biopsy
Euploid (or mosaic)	Euploid or undetectable	May support consideration of transfer
	Non-euploid	May provide less supportive information for transfer
Repeated transfer failures	Euploid	May support consideration of non-embryonic factors
	Non-euploid	May support consideration of embryonic factors

It is important to note that the BRD samples used in this study may contain a considerable proportion of blastocoelic fluid, together with small amounts of other embryo-derived genetic material introduced during the biopsy procedure, including occasional blastocyst cells. Therefore, the processes of BRD collection, DNA amplification, CNV detection, and data analysis require further optimization. Necessary measures should be taken to minimize maternal or exogenous contamination. These measures may include more thorough denudation of blastocysts before biopsy, replacement of the biopsy droplet with a fresh droplet after laser-assisted zona opening, exclusion of washing droplets from sample collection, omission of the lysis step before amplification when appropriate, and incorporation of maternal contamination correction during data analysis. Additionally, this was a single-center study focused on PGT-SR patients, and some analyses were based on relatively small sample sizes. Therefore, the conclusions require broader validation. Overall, although BRD-based genetic analysis remains at an early stage and requires further validation, it may represent a feasible supplementary approach when used as an adjunct to conventional biopsy-based testing. Rather than providing a definitive “0 or 1” judgment on embryo fate, this strategy may help prioritize embryos on the basis of more comprehensive genetic information (16, 53).

## Conclusions

In this study, BRDs generated during routine biopsy procedures were collected for genetic analysis to explore their potential clinical value. Our findings suggest that BRD-based analysis may provide supplementary information in selected scenarios, particularly in the evaluation of repeat-biopsy cases and of embryos identified as euploid by routine testing. Overall, BRD-based genetic testing may represent a potentially useful adjunct to conventional biopsy-based testing in PGT-SR, and further validation is warranted.

## Data Availability

The embryo genetic testing data and associated clinical information presented in this article are not readily available because they contain sensitive human genetic and reproductive health information. Requests to access the datasets should be directed to the correspondence authors.
