# Supplementary material for: Genetic analysis of biopsy-related droplets and its potential applications in PGT-SR patients
Source: Front Endocrinol (Lausanne). 2026 Oct 21;17:1946046. doi: 10.3389/fendo.2026.1946046 (PMC13730871; doi:10.3389/fendo.2026.1946046)
Supplement: Supplementary file 1 [file Table1.docx]

**Supplementary Table 1** CNV Results of Biopsy Cells and Their Corresponding BRDs

| **Patient karyotype** | **Blastocyst code** | **Developmental Day** | **Morphological Grade** | **Biopsy Cells and BRD CNV profiles** | **Consistency** |
| --- | --- | --- | --- | --- | --- |
| 46,XX,t(2;9)(q32.2;p24) | CC1 | D6 | 4BB | Biopsy Cells: Euploid | 2 |
|  |  |  |  |  |  |
|  |  |  |  | BRD: Euploid |  |
|  |  |  |  |  |  |
| 46,XY,t(2;16)(q36;q21) | CC2 | D6 | 6BB | Biopsy Cells: del(2)(q35q37.3)(~22.20Mb),dup(16)(q13q24.3)(~33.15Mb) | 2 |
|  |  |  |  |  |  |
|  |  |  |  | BRD: del(2)(q35q37.3)(~22.20Mb),dup(16)(q13q24.3)(~33.35Mb,~69%) |  |
|  |  |  |  |  |  |
| 46,XX,t(3;4)(p24.3;q21.3) | CC3 | D6 | 4BB | Biopsy Cells: dup(3)(p26.3p24.3)(~23.40Mb), del(4)(q21.21q35.2)(~109.00Mb) | 2 |
|  |  |  |  |  |  |
|  |  |  |  | BRD: dup(3)(p26.3p24.2)(~24.50Mb),del(4)(q21.21q35.2)(~109.15Mb) |  |
|  |  |  |  |  |  |
| 45,XX,der(15;22)(q10;q10) | CC4 | D6 | 4BB | Biopsy Cells: -X,-16 | 2 |
|  |  |  |  |  |  |
|  |  |  |  | BRD: -X(~56%),del(16)(q12.1q24.3)(~41.35Mb,~38%) |  |
|  |  |  |  |  |  |
| 46,XX,inv(9)(p11q13) | CC5 | D6 | 4BA | Biopsy Cells: Euploid | 2 |
|  |  |  |  |  |  |
|  |  |  |  | BRD: Euploid |  |
|  |  |  |  |  |  |
| 46,XY,t(1;3)(q25;p23) | CC6 | D5 | 4BB | Biopsy Cells: -22 | 2 |
|  |  |  |  |  |  |
|  |  |  |  | BRD: -22 |  |
|  |  |  |  |  |  |
| 46,XX,t(3;4)(q26.CC63;q27) | CC7 | D5 | 4BB | Biopsy Cells: dup(3)(q27.1q29)(~14.20Mb),del(4)(q28.1q35.2)(~66.20Mb) | 2 |
|  |  |  |  |  |  |
|  |  |  |  | BRD: dup(3)(q27.1q29)(~14.52Mb),del(4)(q28.1q35.2)(~65.15Mb) |  |
|  |  |  |  |  |  |
| 46,XX,t(3;4)(q26.3;q27) | CC8 | D6 | 4BC | Biopsy Cells: Euploid | 2 |
|  |  |  |  |  |  |
|  |  |  |  | BRD: Euploid |  |
|  |  |  |  |  |  |
| 46,XY,t(2;16)(q36;q21) | CC9 | D5 | 4BB | Biopsy Cells: dup(2)(p25.3q35)(~221.20Mb),del(16)(p13.3q21)(~57.60Mb) | 2 |
|  |  |  |  |  |  |
|  |  |  |  | BRD: dup(2)(p25.3q35)(~221.00Mb),del(16)(p13.3q21)(~57.50Mb) |  |
|  |  |  |  |  |  |
| 46,XX,t(2;9)(q32.2;p24) | CC10 | D5 | 4BB | Biopsy Cells: Euploid | 2 |
|  |  |  |  |  |  |
|  |  |  |  | BRD: Euploid |  |
|  |  |  |  |  |  |
| seq[hg19]del(17)(p12) | CC11 | D6 | 4BC | Biopsy Cells: Euploid | 2 |
|  |  |  |  |  |  |
|  |  |  |  | BRD: Euploid |  |
|  |  |  |  |  |  |
| 46,XY,rob(13;14)(q10;q10) | CC12 | D6 | 5BB | Biopsy Cells: -13 | 2 |
|  |  |  |  |  |  |
|  |  |  |  | BRD: -13 |  |
|  |  |  |  |  |  |
| 46,XX,t(4;20)(q21.2;p11.2) | CC13 | D6 | 4BC | Biopsy Cells: dup(4)(p16.3q13.3)(~72.60Mb),del(20)(p11.21q13.33)(~37.63Mb) | 2 |
|  |  |  |  |  |  |
|  |  |  |  | BRD: dup(4)(p16.3q13.3)(~73.00Mb),del(20)(q11.21q13.33)(~33.53Mb) |  |
|  |  |  |  |  |  |
| 46,XX,t(13;16)(q34;q12) | CC14 | D5 | 4BB | Biopsy Cells: -16 | 2 |
|  |  |  |  |  |  |
|  |  |  |  | BRD: -16 |  |
|  |  |  |  |  |  |
| 46,XX,t(3;6)(q26;q13) | CC15 | D6 | 4BB | Biopsy Cells: del(3)(q26.31q29)(~24.60Mb),dup(6)(q13q27)(~96.52Mb) | 2 |
|  |  |  |  |  |  |
|  |  |  |  | BRD: del(3)(q26.31q29)(~24.52Mb),dup(6)(q13q27)(~96.62Mb) |  |
|  |  |  |  |  |  |
| 46,XX,t(3;6)(q26;q13) | CC16 | D6 | 4BC | Biopsy Cells: del(3)(p26.3q26.31)(~173.60Mb),del(6)(q13q27)(~96.72Mb) | 2 |
|  |  |  |  |  |  |
|  |  |  |  | BRD: del(3)(p26.3q26.31)(~173.50Mb),del(6)(q13q27)(~96.12Mb) |  |
|  |  |  |  |  |  |
| Y chromosome microdeletion | CC17 | D6 | 5AA | Biopsy Cells: Euploid | 2 |
|  |  |  |  |  |  |
|  |  |  |  | BRD: Euploid |  |
|  |  |  |  |  |  |
| Y chromosome microdeletion | CC18 | D6 | 3BC | Biopsy Cells: -16 | 2 |
|  |  |  |  |  |  |
|  |  |  |  | BRD: -16(~63%) |  |
|  |  |  |  |  |  |
| 45,XX,rob(13;15)(q10;q10) | CC19 | D6 | 4BC | Biopsy Cells: Euploid | 2 |
|  |  |  |  |  |  |
|  |  |  |  | BRD: Euploid |  |
|  |  |  |  |  |  |
| 45,XX，der（21；21）（q10；q10） | CC20 | D6 | 4BB | Biopsy Cells: -18,-21 | 2 |
|  |  |  |  |  |  |
|  |  |  |  | BRD: del(18)(p11.32q12.2)(~33.50Mb),del(18)(q12.2q23)(~41.08Mb),-21 |  |
|  |  |  |  |  |  |
| 46,XY,t(6;9)(p21.2;q33) | CC21 | D6 | 3BC | Biopsy Cells: -6,+9,del(12)(q24.23q24.33)(~13.65Mb),-21 | 2 |
|  |  |  |  |  |  |
|  |  |  |  | BRD: -6,+9,del(12)(p13.33q24.23)(~120.50Mb,~41%),del(12)(q24.23q24.33)(~13.85Mb),-21 |  |
|  |  |  |  |  |  |
| 46,XX,t(3;5)(q22;q13.2) | CC22 | D6 | 4BC | Biopsy Cells: del(3)(q25.1q29)(~48.60Mb),dup(5)(q14.3q35.3)(~95.52Mb) | 2 |
|  |  |  |  |  |  |
|  |  |  |  | BRD: del(3)(q25.1q29)(~49.02Mb),dup(5)(q14.3q35.3)(~95.42Mb,~50%) |  |
|  |  |  |  |  |  |
| 45,XY,der(15;22)(q10;q10) | CC23 | D6 | 4BB | Biopsy Cells: Euploid | 2 |
|  |  |  |  |  |  |
|  |  |  |  | BRD: Euploid |  |
|  |  |  |  |  |  |
| 45,XY,der(15;22)(q10;q10) | CC24 | D6 | 3BC | Biopsy Cells: Euploid | 2 |
|  |  |  |  |  |  |
|  |  |  |  | BRD: Euploid |  |
|  |  |  |  |  |  |
| 46,XY,t(12;16)(q14;q23) | CC25 | D6 | 3BB | Biopsy Cells: del(12)(q21.1q24.33)(~61.05Mb),dup(16)(q23.2q24.3)(~10.35Mb) | 2 |
|  |  |  |  |  |  |
|  |  |  |  | BRD: del(12)(q21.1q24.33)(~61.35Mb),dup(16)(q23.2q24.3)(~10.85Mb) |  |
|  |  |  |  |  |  |
| 46,XX,t(3;6)(q26;q13) | CC26 | D6 | 4BB | Biopsy Cells: Euploid | 2 |
|  |  |  |  |  |  |
|  |  |  |  | BRD: Euploid |  |
|  |  |  |  |  |  |
| 46,XY,t(9;17)(q22;p11.2) | CC27 | D6 | 4BB | Biopsy Cells: del(9)(q22.32q34.3)(~44.20Mb),dup(17)(p13.2p11.2)(~15.60Mb) | 2 |
|  |  |  |  |  |  |
|  |  |  |  | BRD: del(9)(q22.32q34.3)(~44.21Mb,~63%),dup(17)(p13.3p11.2)(~22.00Mb,~57%) |  |
|  |  |  |  |  |  |
| 46,XY,t(9;17)(q22;p11.2) | CC28 | D6 | 4BB | Biopsy Cells: Euploid | 2 |
|  |  |  |  |  |  |
|  |  |  |  | BRD: Euploid |  |
|  |  |  |  |  |  |
| 46,XX,t(4;6)(q25;p22.3) | CC29 | D6 | 5BB | Biopsy Cells: del(4)(q25q35.2)(~82.60Mb),dup(6)(p25.3p22.3)(~16.60Mb) | 2 |
|  |  |  |  |  |  |
|  |  |  |  | BRD: del(4)(q25q35.2)(~82.65Mb),dup(6)(p25.3p22.3)(~17.00Mb) |  |
|  |  |  |  |  |  |
| 45,XX,der(14;15)(q10;q10) | CC30 | D6 | 4BC | Biopsy Cells: -22 | 2 |
|  |  |  |  |  |  |
|  |  |  |  | BRD: -22 |  |
|  |  |  |  |  |  |
| 46,XY,t(11;22)(q24.1;q12) | CC31 | D6 | 3BB | Biopsy Cells: +5,dup(12)(p13.33q21.32)(~88.80Mb),del(12)(q23.1q24.33)(~32.45Mb) | 2 |
|  |  |  |  |  |  |
|  |  |  |  | BRD: +5(~63%),dup(12)(p13.33q21.32)(~89.00Mb),dup(12)(q21.32q23.2)(~13.50Mb),del(12)(q23.1q24.33)(~32.35Mb) |  |
|  |  |  |  |  |  |
| 46,XY,t(11;22)(q24.1;q12) | CC32 | D6 | 3BB | Biopsy Cells: del(11)(q23.3q25)(~18.61Mb),dup(22)(q11.21q13.33)(~30.70Mb) | 2 |
|  |  |  |  |  |  |
|  |  |  |  | BRD: del(11)(q23.3q25)(~18.51Mb),dup(22)(q11.21q12.3)(~11.50Mb),dup(22)(q12.3q13.33)(~16.80Mb) |  |
|  |  |  |  |  |  |
| 46,XY,t(2;9)(p14;q34.2) | CC33 | D6 | 4BA | Biopsy Cells: Euploid | 2 |
|  |  |  |  |  |  |
|  |  |  |  | BRD: Euploid |  |
|  |  |  |  |  |  |
| 46,XY,t(2;9)(p14;q34.2) | CC34 | D6 | 4BC | Biopsy Cells: del(2)(p25.3p14)(~65.20Mb),dup(9)(q34.2q34.3)(~4.60Mb),-13,+21 | 2 |
|  |  |  |  |  |  |
|  |  |  |  | BRD: del(2)(p25.3p14)(~66.50Mb,~43%),-13(~31%),+21 |  |
|  |  |  |  |  |  |
| 46,XY,t(2;9)(p14;q34.2) | CC35 | D6 | 4BB | Biopsy Cells: Euploid | 2 |
|  |  |  |  |  |  |
|  |  |  |  | BRD: Euploid |  |
|  |  |  |  |  |  |
| 45,XX,rob(14;21)(q10;q10) | CC36 | D6 | 4BC | Biopsy Cells: Euploid | 2 |
|  |  |  |  |  |  |
|  |  |  |  | BRD: Euploid |  |
|  |  |  |  |  |  |
| 46,XY,der(14;21)(q10;q10) | CC37 | D6 | 4AB | Biopsy Cells: Euploid | 2 |
|  |  |  |  |  |  |
|  |  |  |  | BRD: Euploid |  |
|  |  |  |  |  |  |
| 46,XY,der(14;21)(q10;q10) | CC38 | D6 | 4BB | Biopsy Cells: Euploid | 2 |
|  |  |  |  |  |  |
|  |  |  |  | BRD: Euploid |  |
|  |  |  |  |  |  |
| 46,XX,inv(8)(p12q12) | CC39 | D6 | 4BB | Biopsy Cells: Euploid | 2 |
|  |  |  |  |  |  |
|  |  |  |  | BRD: Euploid |  |
|  |  |  |  |  |  |
| 46,XY,t(13;15)(q22;q14) | CC40 | D5 | 4AB | Biopsy Cells: Euploid | 2 |
|  |  |  |  |  |  |
|  |  |  |  | BRD: Euploid |  |
|  |  |  |  |  |  |
| 46,XX,t(4;20)(p10;p10) | CC41 | D5 | 4AB | Biopsy Cells: Euploid | 2 |
|  |  |  |  |  |  |
|  |  |  |  | BRD: Euploid |  |
|  |  |  |  |  |  |
| 46,XY,t(3;5)(q22;q13.2) | CC42 | D5 | 4BB | Biopsy Cells: Euploid | 2 |
|  |  |  |  |  |  |
|  |  |  |  | BRD: Euploid |  |
|  |  |  |  |  |  |
| 46,XY,t(3;5)(q22;q13.2) | CC43 | D5 | 4BB | Biopsy Cells: Euploid | 2 |
|  |  |  |  |  |  |
|  |  |  |  | BRD: Euploid |  |
|  |  |  |  |  |  |
| 46,XY,t(3;5)(q22;q13.2) | CC44 | D6 | 4BB | Biopsy Cells: Euploid | 2 |
|  |  |  |  |  |  |
|  |  |  |  | BRD: Euploid |  |
|  |  |  |  |  |  |
| 46,XY,t(3;5)(q22;q13.2) | CC45 | D6 | 4BC | Biopsy Cells: Euploid | 2 |
|  |  |  |  |  |  |
|  |  |  |  | BRD: Euploid |  |
|  |  |  |  |  |  |
| 46,XY,t(3;5)(q22;q13.2) | CC46 | D6 | 5BB | Biopsy Cells: Euploid | 2 |
|  |  |  |  |  |  |
|  |  |  |  | BRD: Euploid |  |
|  |  |  |  |  |  |
| 46,XX,t(2;14)(p21;q32.1) | CC47 | D6 | 4BC | Biopsy Cells: del(2)(p25.3p21)(~43.00Mb),dup(14)(q32.2q32.33)(~10.75Mb) | 2 |
|  |  |  |  |  |  |
|  |  |  |  | BRD: del(2)(p25.3p21)(~43.00Mb,~55%),dup(14)(q32.2q32.33)(~10.85Mb,~56%) |  |
|  |  |  |  |  |  |
| 46,XX,t(2;14)(p21;q32.1) | CC48 | D6 | 4BC | Biopsy Cells: del(2)(p25.3p21)(~43.00Mb),dup(14)(q32.2q32.33)(~10.95Mb),+22 | 2 |
|  |  |  |  |  |  |
|  |  |  |  | BRD: del(2)(p25.3p21)(~42.00Mb,~65%),dup(14)(q32.2q32.33)(~10.85Mb),+22(~69%) |  |
|  |  |  |  |  |  |
| 46，XX，t（2: 14）（p21:q32.1） | CC49 | D6 | 4BC | Biopsy Cells: dup(2)(p21q37.3)(~200.40Mb),del(14)(q11.2q32.2)(~76.80Mb),+19 | 2 |
|  |  |  |  |  |  |
|  |  |  |  | BRD: dup(2)(p21q37.3)(~200.70Mb),del(14)(q11.2q32.2)(~76.50Mb,~68%),dup(19)(p13.3q13.33)(~49.50Mb,~56%) |  |
|  |  |  |  |  |  |
| 45,XY,rob(13;14)(q10;q10) | CC50 | D6 | 4BB | Biopsy Cells: Euploid | 2 |
|  |  |  |  |  |  |
|  |  |  |  | BRD: Euploid |  |
|  |  |  |  |  |  |
| 45,XY,rob(13;14)(q10;q10) | CC51 | D6 | 5CB | Biopsy Cells: Euploid | 2 |
|  |  |  |  |  |  |
|  |  |  |  | BRD: Euploid |  |
|  |  |  |  |  |  |
| 46,XX,inv(8)(q12q24.2) | CC52 | D6 | 4BB | Biopsy Cells: -8 | 2 |
|  |  |  |  |  |  |
|  |  |  |  | BRD: -8 |  |
|  |  |  |  |  |  |
| 46,XY,t(5:7)(p15.1;p13) | CC53 | D6 | 4AA | Biopsy Cells: del(5)(p15.33p14.3)(~21.80Mb),dup(7)(p22.3p11.2)(~55.20Mb) | 2 |
|  |  |  |  |  |  |
|  |  |  |  | BRD: del(5)(p15.33p14.3)(~22.00Mb),dup(7)(p22.3p11.2)(~55.50Mb) |  |
|  |  |  |  |  |  |
| 45,XX,rob(14;21)(q10;q10) | CC54 | D6 | 4BC | Biopsy Cells: -14 | 2 |
|  |  |  |  |  |  |
|  |  |  |  | BRD: -14 |  |
|  |  |  |  |  |  |
| 46,XY,t(1;4)(q25;q24.3) | CC55 | D6 | 4BB | Biopsy Cells: Euploid | 2 |
|  |  |  |  |  |  |
|  |  |  |  | BRD: Euploid |  |
|  |  |  |  |  |  |
| 46,XY,t(1;4)(q25;q24.3) | CC56 | D6 | 4BC | Biopsy Cells: Euploid | 2 |
|  |  |  |  |  |  |
|  |  |  |  | BRD: Euploid |  |
|  |  |  |  |  |  |
| 45,XX,rob(13;15)(q10;q10) | CC57 | D6 | 4BC | Biopsy Cells: +16 | 2 |
|  |  |  |  |  |  |
|  |  |  |  | BRD: +16(~66%) |  |
|  |  |  |  |  |  |
| 46,XX,inv(8)(q12;q24.2) | CC58 | D5 | 4BB | Biopsy Cells: -15,+16 | 2 |
|  |  |  |  |  |  |
|  |  |  |  | BRD: -15,+16 |  |
|  |  |  |  |  |  |
| 45,XX,rob(14;21)(q10;q10) | PC1 | D5 | 4BB | Biopsy Cells: -21,-22 | 1 |
|  |  |  |  |  |  |
|  |  |  |  | BRD: +1(~47%),-21,-22(~57%) |  |
|  |  |  |  |  |  |
| 46,XY,t(10;17)(p13;q11.2) | PC2 | D5 | 4BB | Biopsy Cells: del(10)(p15.3p15.2)(~3.80Mb),dup(17)(p13.3p13.1)(~10.40Mb) | 1 |
|  |  |  |  |  |  |
|  |  |  |  | BRD: dup(17)(p13.3p12)(~11.00Mb) |  |
|  |  |  |  |  |  |
| 46,XX,t(3;4)(p24.3;q21.3) | PC3 | D6 | 4BC | Biopsy Cells: -2(~56%), dup(3)(p26.3p24.3)(~23.60Mb), del(4)(q21.21q35.2)(~109.40Mb) | 1 |
|  |  |  |  |  |  |
|  |  |  |  | BRD: dup(3)(p26.3p24.2)(~24.00Mb),dup(4)(p16.3q21.21)(~82.00Mb,~35%),del(4)(q21.21q35.2)(~109.65Mb),-9(~50%) |  |
|  |  |  |  |  |  |
| 46,XY,t(2;16)(q36;q21) | PC4 | D6 | 4BC | Biopsy Cells: -21 | 1 |
|  |  |  |  |  |  |
|  |  |  |  | BRD: dup(9)(p24.3p13.1)(~39.00Mb,~35%),del(9)(q21.11q34.3)(~70.71Mb,~48%),-21 |  |
|  |  |  |  |  |  |
| 46,XY,t(2;16)(q36;q21) | PC5 | D6 | 4BB | Biopsy Cells: dup(2)(q35q37.3)(~22.20Mb),-15,del(16)(q13q24.3)(~33.15Mb) | 1 |
|  |  |  |  |  |  |
|  |  |  |  | BRD: dup(2)(q34q37.3)(~28.20Mb),+7(~39%),-15(~68%),del(16)(q13q24.3)(~33.35Mb) |  |
|  |  |  |  |  |  |
| 46,XX,t(2;9)(q32.2;p24) | PC6 | D5 | 4BB | Biopsy Cells: dup(2)(q32.2q37.3)(~52.00Mb),del(9)(p24.3p24.1)(~4.80Mb) | 1 |
|  |  |  |  |  |  |
|  |  |  |  | BRD: dup(2)(q32.3q37.3)(~46.70Mb,~54%) |  |
|  |  |  |  |  |  |
| 46,XX,t(2;9)(q32.2;p24) | PC7 | D5 | 4BB | Biopsy Cells: del(2)(p25.3q32.2)(~191.40Mb),del(9)(p24.3p24.2)(~4.60Mb) | 1 |
|  |  |  |  |  |  |
|  |  |  |  | BRD: del(2)(p25.3q32.3)(~192.00Mb,~31%) |  |
|  |  |  |  |  |  |
| 46,XX,t(2;9)(q32.2;p24) | PC8 | D6 | 4BB | Biopsy Cells: del(6)(p25.3p21.2)(~39.60Mb),del(6)(p21.2q27)(~131.72Mb,~36%) | 1 |
|  |  |  |  |  |  |
|  |  |  |  | BRD: del(1)(p36.33p34.1)(~46.00Mb,~35%),del(6)(p25.3p21.2)(~38.50Mb),del(9)(q31.1q33.3)(~18.00Mb,~63%) |  |
|  |  |  |  |  |  |
| 46,XY,rob(13;14)(q10;q10) | PC9 | D6 | 4BB | Biopsy Cells: +9(~31%),+14 | 1 |
|  |  |  |  |  |  |
|  |  |  |  | BRD: +3,dup(6)(p25.3q25.3)(~158.00Mb),-12(~66%),+14,+16 |  |
|  |  |  |  |  |  |
| 46,XY,rob(13;14)(q10;q10) | PC10 | D6 | 4BC | Biopsy Cells: +4(~46%),+14,+16 | 1 |
|  |  |  |  |  |  |
|  |  |  |  | BRD: -4(~49%),dup(9)(p24.3p21.1)(~33.00Mb,~61%),+14,+16,dup(18)(q12.1q23)(~47.08Mb,~33%),dup(19)(q11q13.43)(~31.13Mb) |  |
|  |  |  |  |  |  |
| 46,XY,t(10,17)(p13;p11.2) | PC11 | D6 | 4BC | Biopsy Cells: dup(10)(p15.3p15.2)(~3.80Mb),del(17)(p13.3p13.1)(~10.40Mb) | 1 |
|  |  |  |  |  |  |
|  |  |  |  | BRD: del(17)(p13.3p12)(~13.50Mb) |  |
|  |  |  |  |  |  |
| 46,XY,t(14;16)(q32;q13) | PC12 | D5 | 4BB | Biopsy Cells: dup(14)(q31.3q32.33)(~18.35Mb),del(16)(q12.1q24.3)(~39.75Mb),-18 | 1 |
|  |  |  |  |  |  |
|  |  |  |  | BRD: -1(~54%),del(2)(q32.3q37.1)(~35.50Mb),dup(8)(p23.3q12.3)(~65.50Mb),dup(8)(q24.22q24.3)(~13.86Mb),del(9)(p24.3q33.2)(~123.50Mb),+13,+14,dup(16)(p13.3q12.1)(~51.00Mb,~31%),del(16)(q12.1q24.3)(~39.85Mb,~57%),-17(~66%),-18,+20,+22(~46%) |  |
|  |  |  |  |  |  |
| 46,XX,t(11;22)(q24.2;q13.1) | PC13 | D5 | 4BC | Biopsy Cells: del(11)(p15.5q23.3)(~117.00Mb),dup(22)(q11.1q11.21)(~3.20Mb) | 1 |
|  |  |  |  |  |  |
|  |  |  |  | BRD: del(11)(p15.5q23.3)(~117.00Mb),dup(11)(q23.3q25)(~18.21Mb,~37%) |  |
|  |  |  |  |  |  |
| 45,XX,der(21;21)(q10;q10) | PC14 | D6 | 4BB | Biopsy Cells: -17(~41%),-21 | 1 |
|  |  |  |  |  |  |
|  |  |  |  | BRD: -5(~64%),-7(~49%),-13(~67%),-16(~41%),+18,-21 |  |
|  |  |  |  |  |  |
| 46,XX,t(2;4)(p16;q27) | PC15 | D6 | 4BC | Biopsy Cells: dup(2)(p16.1q37.3)(~182.20Mb),del(4)(p16.3q28.2)(~129.00Mb) | 1 |
|  |  |  |  |  |  |
|  |  |  |  | BRD: dup(2)(p16.1q37.3)(~182.20Mb),del(4)(p16.3q28.2)(~129.50Mb),+7(~45%),+8,+9(~57%),dup(10)(q11.21q26.3)(~92.53Mb,~64%),+16(~42%),+17(~47%),-21(~44%) |  |
|  |  |  |  |  |  |
| 46,XY,t(6;10)(p24.3;p15.1) | PC16 | D6 | 4BB | Biopsy Cells: dup(6)(p25.3p24.3)(~7.40Mb),dup(8)(p23.2)(~2.40Mb), del(10)(p15.3p15.1)(~5.40Mb), dup(16)(p13.11)(~1.00Mb) | 1 |
|  |  |  |  |  |  |
|  |  |  |  | BRD: +X(~32%),dup(6)(p25.3p25.1)(~7.00Mb),del(10)(p15.3p15.1)(~5.20Mb) |  |
|  |  |  |  |  |  |
| Y chromosome microdeletion | PC17 | D6 | 6BB | Biopsy Cells: del(X)(p22.33)(~1.20Mb),+18 | 1 |
|  |  |  |  |  |  |
|  |  |  |  | BRD: +18 |  |
|  |  |  |  |  |  |
| 46,XY,t(6;9)(p21.2;q33) | PC18 | D6 | 4BB | Biopsy Cells: dup(6)(p25.3p21.33)(~31.00Mb),del(9)(q34.11q34.3)(~9.00Mb) | 1 |
|  |  |  |  |  |  |
|  |  |  |  | BRD: dup(6)(p25.3p21.33)(~30.50Mb) |  |
|  |  |  |  |  |  |
| 46,XY,t(6;9)(p21.2;q33) | PC19 | D6 | 4BC | Biopsy Cells: +5,dup(6)(p25.3p21.33)(~30.60Mb),del(9)(q34.11q34.3)(~9.00Mb) | 1 |
|  |  |  |  |  |  |
|  |  |  |  | BRD: +5(~70%),dup(6)(p25.3p11.2)(~58.00Mb,~70%) |  |
|  |  |  |  |  |  |
| 46,XX,t(3;5)(q22;q13.2) | PC20 | D6 | 4BC | Biopsy Cells: dup(3)(q25.1q29)(~48.60Mb),del(5)(q14.3q35.3)(~95.72Mb) | 1 |
|  |  |  |  |  |  |
|  |  |  |  | BRD: dup(3)(q25.1q26.2)(~21.00Mb),dup(3)(q26.31q29)(~24.52Mb),del(5)(q14.3q35.3)(~95.92Mb),dup(17)(p13.3p13.1)(~10.00Mb) |  |
|  |  |  |  |  |  |
| 46,XX,t(3;6)(q26;q13) | PC21 | D6 | 4BB | Biopsy Cells: +3,-6,-18(~31%) | 1 |
|  |  |  |  |  |  |
|  |  |  |  | BRD: +2,+3,-6(~69%),-16(~69%) |  |
|  |  |  |  |  |  |
| 45,XX,der(15;22)(q10;q10) | PC22 | D5 | 4AB | Biopsy Cells: dup(X)(q25q28)(~28.07Mb),-15 | 1 |
|  |  |  |  |  |  |
|  |  |  |  | BRD: -15 |  |
|  |  |  |  |  |  |
| 46,XY,t(2:20)(q14.1;q13.2) | PC23 | D6 | 4BB | Biopsy Cells: dup(2)(q11.2q37.3)(~143.80Mb),del(20)(q11.23q13.33)(~26.23Mb) | 1 |
|  |  |  |  |  |  |
|  |  |  |  | BRD: dup(2)(q11.1q37.3)(~148.20Mb,~36%) |  |
|  |  |  |  |  |  |
| 46,XY,t(9;17)(q22;p11.2) | PC24 | D6 | 4BB | Biopsy Cells: del(9)(q22.32q34.3)(~42.20Mb),dup(17)(p13.3p11.2)(~20.40Mb) | 1 |
|  |  |  |  |  |  |
|  |  |  |  | BRD: del(9)(q22.32q34.3)(~44.21Mb,~60%),-10(~38%),dup(17)(p13.3p11.2)(~22.00Mb) |  |
|  |  |  |  |  |  |
| 46,XY,t(9;17)(q22;p11.2) | PC25 | D6 | 4BB | Biopsy Cells: dup(8)(p23.3p11.23)(~36.40Mb),dup(9)(q22.32q34.2)(~39.60Mb),dup(9)(q34.2q34.3)(~4.80Mb),+15(~46%),-16,del(17)(p13.3p11.2)(~20.40Mb) | 1 |
|  |  |  |  |  |  |
|  |  |  |  | BRD: dup(9)(q22.32q34.3)(~43.21Mb,~54%),-16(~43%),del(17)(p13.3p11.2)(~22.00Mb,~44%) |  |
|  |  |  |  |  |  |
| 46,XY,t(9;17)(q22;p11.2) | PC26 | D6 | 4BB | Biopsy Cells: del(9)(q22.32q34.3)(~44.20Mb),-16(~33%),dup(17)(p13.3p11.2)(~20.40Mb) | 1 |
|  |  |  |  |  |  |
|  |  |  |  | BRD: del(9)(q22.31q34.3)(~44.71Mb),dup(17)(p13.3p11.2)(~22.00Mb) |  |
|  |  |  |  |  |  |
| 46,XY,t(9;17)(q22;p11.2) | PC27 | D6 | 4BB | Biopsy Cells: dup(9)(q22.32q34.3)(~44.20Mb),del(17)(p13.3p11.2)(~21.00Mb) | 1 |
|  |  |  |  |  |  |
|  |  |  |  | BRD: dup(9)(q22.31q34.3)(~45.21Mb) |  |
|  |  |  |  |  |  |
| 46,XX,t(4;6)(q25;p22.3) | PC28 | D6 | 4BB | Biopsy Cells: dup(4)(q25q35.2)(~82.40Mb),del(6)(p25.3p22.3)(~16.20Mb) | 1 |
|  |  |  |  |  |  |
|  |  |  |  | BRD: dup(4)(q25q35.2)(~82.65Mb,~68%),del(6)(p25.3p22.3)(~16.50Mb),del(7)(q21.13q36.3)(~68.14Mb,~56%) |  |
|  |  |  |  |  |  |
| 46,XY,t(11;22)(q24.1;q12) | PC29 | D5 | 4BB | Biopsy Cells: +2(~51%),+12(~39%) | 1 |
|  |  |  |  |  |  |
|  |  |  |  | BRD: -1,+2,-4,+5,-8,-11,+12,-14,-19(~69%),+20 |  |
|  |  |  |  |  |  |
| 46,XY,t(11;22)(q24.1;q12) | PC30 | D6 | 4BB | Biopsy Cells: dup(11)(q23.3q25)(~18.41Mb),del(14)(q21.2q32.33)(~62.35Mb),del(22)(q11.21q13.33)(~30.70Mb) | 1 |
|  |  |  |  |  |  |
|  |  |  |  | BRD: dup(11)(q23.3q25)(~18.51Mb),dup(14)(q21.3q32.33)(~57.35Mb,~47%),del(22)(q11.21q13.33)(~30.80Mb) |  |
|  |  |  |  |  |  |
| 46,XY,t(2;9)(p14;q34.2) | PC31 | D6 | 4BC | Biopsy Cells: del(2)(p14q37.3)(~178.20Mb),+9,-16 | 1 |
|  |  |  |  |  |  |
|  |  |  |  | BRD: del(2)(p14q37.3)(~178.20Mb,~35%) |  |
|  |  |  |  |  |  |
| 46,XX,t(9;15)(q32;q23) | PC32 | D6 | 4BC | Biopsy Cells: +1(~35%),-6,del(9)(p24.3q32)(~115.60Mb,~31%),del(9)(q32q34.3)(~25.80Mb),dup(13)(q32.3q33.2)(~4.80Mb),dup(15)(q23q26.3)(~33.53Mb) | 1 |
|  |  |  |  |  |  |
|  |  |  |  | BRD: dup(1)(q32.2q44)(~40.75Mb),-3(~55%),-4(~40%),-6,+7(~41%),del(9)(q32q34.3)(~26.21Mb),+13(~47%),dup(15)(q23q26.3)(~32.03Mb),+17(~60%),+18(~67%) |  |
|  |  |  |  |  |  |
| 45,XX,rob(14;21)(q10;q10) | PC33 | D5 | 4BA | Biopsy Cells: dup(2)(p25.3p11.2)(~89.40Mb,~34%),del(2)(q11.1q37.3)(~147.80Mb,~36%),+3(~32%),+22(~35%) | 1 |
|  |  |  |  |  |  |
|  |  |  |  | BRD: +X,dup(8)(p23.3p11.23)(~37.50Mb),dup(8)(q24.22q24.3)(~14.86Mb),dup(22)(q12.3q13.33)(~15.30Mb) |  |
|  |  |  |  |  |  |
| 46,XX,t(1;13)(q43;q12.2) | PC34 | D6 | 4BC | Biopsy Cells: del(1)(q44)(~4.45Mb),dup(13)(q12.13q34)(~88.57Mb) | 1 |
|  |  |  |  |  |  |
|  |  |  |  | BRD: +3(~45%),+11(~47%),+13(~47%) |  |
|  |  |  |  |  |  |
| 46,XY,der(14;21)(q10;q10) | PC35 | D6 | 4BB | Biopsy Cells: +21,-22 | 1 |
|  |  |  |  |  |  |
|  |  |  |  | BRD: dup(2)(p25.3q24.2)(~161.50Mb,~32%),+21,-22(~69%) |  |
|  |  |  |  |  |  |
| 46,XY,t(13;15)(q22;q14) | PC36 | D6 | 4BC | Biopsy Cells: -4,-15(~51%) | 1 |
|  |  |  |  |  |  |
|  |  |  |  | BRD: +X(~35%),-Y(~50%),dup(9)(p24.3p13.3)(~33.50Mb),dup(11)(p15.5p15.1)(~16.50Mb),dup(11)(p15.2p14.3)(~10.00Mb),dup(13)(q11q12.3)(~11.50Mb),dup(13)(q12.3q14.2)(~17.50Mb),dup(13)(q14.3q21.31)(~11.50Mb),dup(13)(q21.33q31.1)(~11.00Mb) |  |
|  |  |  |  |  |  |
| 46,XY,t(10;21)(p12;q22.1) | PC37 | D6 | 4BC | Biopsy Cells: del(10)(p12.2q26.3)(~112.33Mb),dup(21)(q11.2q21.3)(~14.80Mb) | 1 |
|  |  |  |  |  |  |
|  |  |  |  | BRD: +X(~59%),+1,+3(~40%),+4(~63%),-6(~53%),dup(10)(p15.3p12.2)(~23.50Mb,~39%),del(10)(p12.2q26.3)(~112.53Mb),-12,dup(17)(p13.3p11.2)(~22.00Mb),-18,-20(~53%),dup(21)(q11.2q21.3)(~14.50Mb),del(21)(q21.3q22.3)(~19.13Mb,~35%),+22(~40%) |  |
|  |  |  |  |  |  |
| 46,XX,t(4;20)(q21.2;p11.2) | PC38 | D6 | 4BB | Biopsy Cells: dup(4)(q13.3q35.2)(~118.20Mb),del(20)(p13p11.1)(~26.00Mb) | 1 |
|  |  |  |  |  |  |
|  |  |  |  | BRD: dup(4)(q13.3q35.2)(~119.15Mb),-7(~56%),dup(12)(p13.33p11.1)(~34.50Mb,~62%),dup(17)(p13.3q21.33)(~47.50Mb,~31%),del(20)(p13p11.1)(~26.00Mb),dup(21)(q21.2q22.3)(~24.13Mb,~37%) |  |
|  |  |  |  |  |  |
| 46,XX,inv(8)(q12q24.2) | PC39 | D6 | 4BB | Biopsy Cells: -11(~33%),-16,dup(20)(p13p11.23)(~20.40Mb,~57%) | 1 |
|  |  |  |  |  |  |
|  |  |  |  | BRD: -16 |  |
|  |  |  |  |  |  |
| 46,XX,t(3;12)(q23;q15) | PC40 | D6 | 4BB | Biopsy Cells: del(3)(q25.32q29)(~41.00Mb),dup(12)(q21.32q24.33)(~46.05Mb),del(19)(q13.2q13.43)(~17.53Mb,~41%) | 1 |
|  |  |  |  |  |  |
|  |  |  |  | BRD: del(3)(q25.32q29)(~41.02Mb),+5(~56%),del(6)(q21q27)(~65.62Mb),-9,+11(~41%),dup(12)(q21.32q24.33)(~46.35Mb),-13(~54%),+21(~48%) |  |
|  |  |  |  |  |  |
| 46,XX,t(3;12)(q23;q15) | PC41 | D6 | 4BB | Biopsy Cells: -6 | 1 |
|  |  |  |  |  |  |
|  |  |  |  | BRD: -3(~66%),-6,-7(~50%),+9(~49%),+10(~55%),+12,+16(~47%),+17(~46%),+18,+21(~42%) |  |
|  |  |  |  |  |  |
| 46,XX,t(3;12)(q23;q15) | PC42 | D6 | 4BC | Biopsy Cells: +1(~59%),-2(~38%),del(3)(q25.32q29)(~41.00Mb),+6(~41%),+7(~36%),-8(~39%),+9(~38%),dup(12)(q21.32q24.33)(~45.85Mb,~56%),dup(13)(q11q22.3)(~58.40Mb),del(13)(q22.3q34)(~36.97Mb),-15(~56%),-19(~52%),+22(~56%) | 1 |
|  |  |  |  |  |  |
|  |  |  |  | BRD: del(3)(q25.32q29)(~41.02Mb),dup(12)(q21.32q24.33)(~45.50Mb),del(13)(q14.11q22.3)(~33.50Mb),dup(13)(q22.3q34)(~37.67Mb,~68%) |  |
|  |  |  |  | PC44 |  |
| 46,XX,t(3;12)(q23;q15) | PC43 | D6 | 4BB | Biopsy Cells: dup(3)(p26.3q25.32)(~157.20Mb,~35%),del(3)(q25.32q29)(~41.00Mb),dup(12)(q21.32q24.33)(~46.05Mb) | 1 |
|  |  |  |  |  |  |
|  |  |  |  | BRD: -1(~32%),del(3)(q25.32q29)(~41.02Mb),dup(12)(q21.32q24.33)(~46.35Mb),-14(~30%) |  |
|  |  |  |  |  |  |
| 45,XX,rob(14;21)(q10;q10) | PC44 | D5 | 4AB | Biopsy Cells: -14 | 1 |
|  |  |  |  |  |  |
|  |  |  |  | BRD: -14,del(19)(p13.2q13.43)(~52.13Mb,~56%) |  |
|  |  |  |  |  |  |
| 45,XX,rob(14;21)(q10;q10) | PC45 | D5 | 4BB | Biopsy Cells: +15,-21 | 1 |
|  |  |  |  |  |  |
|  |  |  |  | BRD: +6(~53%),+7(~45%),-11(~47%),-12(~65%),+15,-16(~42%),+17(~51%),-21 |  |
|  |  |  |  |  |  |
| 46,XY,t(1;14)(q25;q24.3) | PC46 | D5 | 4BB | Biopsy Cells: dup(1)(q31.1q44)(~60.40Mb),del(14)(q31.3q32.33)(~18.15Mb) | 1 |
|  |  |  |  |  |  |
|  |  |  |  | BRD: dup(1)(q31.1q44)(~62.75Mb,~39%),-4(~55%),dup(5)(q31.3q35.1)(~29.50Mb),-14,dup(15)(q26.1q26.3)(~12.03Mb),-16(~59%) |  |
|  |  |  |  |  |  |
| 46,XY,t(1;4)(q25;q24.3) | PC47 | D6 | 4BC | Biopsy Cells: dup(1)(q31.1q44)(~61.25Mb),del(14)(q31.3q32.33)(~18.15Mb) | 1 |
|  |  |  |  |  |  |
|  |  |  |  | BRD: dup(1)(p36.33q31.1)(~187.00Mb,~40%),dup(1)(q31.1q44)(~62.25Mb) |  |
|  |  |  |  |  |  |
| 46,XY,t(1;4)(q25;q24.3) | PC48 | D6 | 4BC | Biopsy Cells: dup(1)(q22q44)(~93.45Mb,~33%),-5(~39%),+14(~33%) | 1 |
|  |  |  |  |  |  |
|  |  |  |  | BRD: del(5)(p15.33p14.3)(~19.50Mb),del(5)(q11.1q35.3)(~131.42Mb),dup(9)(p21.3p13.3)(~11.50Mb),dup(9)(q21.2q34.11)(~51.00Mb),dup(10)(p12.1p11.1)(~13.50Mb),dup(11)(p15.3p14.3)(~13.00Mb),dup(11)(p13p11.12)(~13.50Mb),dup(11)(q14.1q22.3)(~26.00Mb),dup(12)(p12.3p11.21)(~12.00Mb),dup(12)(q11q13.11)(~11.00Mb),dup(12)(q15q24.11)(~39.50Mb),dup(12)(q24.11q24.33)(~24.85Mb),dup(16)(p13.2p12.2)(~14.50Mb),dup(16)(q11.2q21)(~14.00Mb),dup(16)(q22.1q24.1)(~19.00Mb) |  |
|  |  |  |  |  |  |
| 46,XX,t(6;22)(q26;q21.1) | PC49 | D6 | 4BC | Biopsy Cells: dup(6)(q26q27)(~8.72Mb),del(22)(q11.21q13.33)(~30.70Mb) | 1 |
|  |  |  |  |  |  |
|  |  |  |  | BRD: del(3)(p26.3q13.32)(~119.00Mb,~35%),dup(8)(q12.1q24.3)(~89.36Mb),dup(11)(p15.5p11.12)(~49.00Mb),del(22)(q11.21q13.33)(~30.80Mb) |  |
|  |  |  |  |  |  |
| 46,XX,t(6;22)(q26;q21.1) | PC50 | D6 | 4BC | Biopsy Cells: del(6)(q26q27)(~8.72Mb),dup(22)(q11.21q13.33)(~30.70Mb) | 1 |
|  |  |  |  |  |  |
|  |  |  |  | BRD: +22(~40%) |  |
|  |  |  |  |  |  |
| 46,XX,t(4;16)(q26;q23) | PC51 | D6 | 4BC | Biopsy Cells: -3 | 1 |
|  |  |  |  |  |  |
|  |  |  |  | BRD: -3,+8(~42%),-12(~69%),+15(~43%),-17(~62%) |  |
|  |  |  |  |  |  |
| 46,XX,t(4;16)(q26;q23) | PC52 | D6 | 3BC | Biopsy Cells: dup(4)(q25q35.2)(~78.20Mb),del(16)(q22.2q24.3)(~17.75Mb),-20 | 1 |
|  |  |  |  |  |  |
|  |  |  |  | BRD: -3,dup(4)(q25q35.2)(~80.15Mb),-5,-7,-11,-12(~69%),del(16)(q22.2q24.3)(~18.35Mb),-17(~69%),-20,-21,-22 |  |
|  |  |  |  |  |  |
| 45,XY,rob(13;14)(q10;q10) | PC53 | D6 | 4BB | Biopsy Cells: +14 | 1 |
|  |  |  |  |  |  |
|  |  |  |  | BRD: del(2)(q32.2q36.3)(~39.00Mb),del(11)(q22.1q23.3)(~19.50Mb,~70%),del(13)(q33.1q34)(~11.67Mb),+14,del(18)(q21.31q23)(~17.50Mb) |  |
|  |  |  |  |  |  |
| 45,XY,der(13;22)(q10;q10) | PC54 | D5 | 4BB | Biopsy Cells: -16 | 1 |
|  |  |  |  |  |  |
|  |  |  |  | BRD: -X(~52%),+2p(~33%),-2q(~45%),+3,+6p(~66%),+6q,+11q,+14(~55%),-16(~61%) |  |
|  |  |  |  |  |  |
| 45,XY,der(13;22)(q10;q10) | PC55 | D6 | 4BC | Biopsy Cells: +13 | 1 |
|  |  |  |  |  |  |
|  |  |  |  | BRD: del(1)(p36.33p36.21)(~15.00Mb),+13 |  |
|  |  |  |  |  |  |
| 46,XY,t(13;15)(q22;q14) | CI1 | D5 | 4BB | Biopsy Cells: Euploid | 0 |
|  |  |  |  |  |  |
|  |  |  |  | BRD: -14(~43%) |  |
|  |  |  |  |  |  |
| 46,XX,t(3;12)(q23;q15) | CI2 | D6 | 4BB | Biopsy Cells: dup(10)(q11.21q26.3)(~92.53Mb,~33%) | 0 |
|  |  |  |  |  |  |
|  |  |  |  | BRD: del(7)(q22.1q36.3)(~55.64Mb,~41%) |  |
|  |  |  |  |  |  |
| 45,XX,der(15;22)(q10;q10) | CI3 | D6 | 4BA | Biopsy Cells: Euploid | 0 |
|  |  |  |  |  |  |
|  |  |  |  | BRD: -2(~49%),+4,-6,+18,+22(~49%) |  |
|  |  |  |  |  |  |
| 45,XX,der(15;22)(q10;q10) | CI4 | D6 | 4CB | Biopsy Cells: del(7)(q11.21q36.3)(~95.94Mb,~27%) | 0 |
|  |  |  |  |  |  |
|  |  |  |  | BRD: +7(~43%),+15(~54%) |  |
|  |  |  |  |  |  |
| 46,XY,t(2;16)(q36;q21) | CI5 | D6 | 4BB | Biopsy Cells: -4(~36%),+5(~33%),dup(10)(p15.3q21.1)(~56.20Mb,~67%) | 0 |
|  |  |  |  |  |  |
|  |  |  |  | BRD: dup(1)(p36.33p31.1)(~75.00Mb) |  |
|  |  |  |  |  |  |
| 46,XX,t(2;9)(q32.2;p24) | CI6 | D5 | 4BC | Biopsy Cells: +8(~52%),+12(~51%) | 0 |
|  |  |  |  |  |  |
|  |  |  |  | BRD: +X(~43%) |  |
|  |  |  |  |  |  |
| 46,XY,t(10,17)(p13;p11.2) | CI7 | D6 | 4CB | Biopsy Cells: dup(10)(p15.3p15.2)(~3.80Mb),del(17)(p13.3p13.1)(~10.40Mb) | 0 |
|  |  |  |  |  |  |
|  |  |  |  | BRD: dup(11)(p15.5p15.3)(~11.00Mb),+22 |  |
|  |  |  |  |  |  |
| 46,XY,t(6;9)(p21.2;q33) | CI8 | D5 | 4BB | Biopsy Cells: Euploid | 0 |
|  |  |  |  |  |  |
|  |  |  |  | BRD: -2(~31%), dup(3)(p26.3p24.3)(~23.40Mb),del(4)(q21.21q35.2)(~109.40Mb), -10(~32%) |  |
|  |  |  |  |  |  |
| 45,XY,der(15;22)(q10;q10) | CI9 | D6 | 4BB | Biopsy Cells: Euploid | 0 |
|  |  |  |  |  |  |
|  |  |  |  | BRD: +X(~40%),-Y(~30%),dup(4)(p16.3q21.3)(~88.00Mb),del(4)(q21.3q35.2)(~103.65Mb,~56%) |  |
|  |  |  |  |  |  |
| 45,XY,der(15;22)(q10;q10) | CI10 | D6 | 4BC | Biopsy Cells: -13(~34%),+15 | 0 |
|  |  |  |  |  |  |
|  |  |  |  | BRD: -2(~43%) |  |
|  |  |  |  |  |  |
| 45,XX, der(15;22)(q10;q10) | CI11 | D5 | 4BB | Biopsy Cells: Euploid | 0 |
|  |  |  |  |  |  |
|  |  |  |  | BRD: -5(~40%),+17(~54%),dup(20)(q11.21q13.33)(~33.53Mb) |  |
|  |  |  |  |  |  |
| 45,XX, der(15;22)(q10;q10) | CI12 | D6 | 3BC | Biopsy Cells: del(12)(p13.33p12.3)(~16.60Mb,~61%) | 0 |
|  |  |  |  |  |  |
|  |  |  |  | BRD: +21 |  |
|  |  |  |  |  |  |
| 46,XY,t(9;17)(q22;p11.2) | CI13 | D6 | 4BC | Biopsy Cells: +10(~56%),+14(~63%),-22(~39%) | 0 |
|  |  |  |  |  |  |
|  |  |  |  | BRD: Euploid |  |
|  |  |  |  |  |  |
| 46,XY,t(2;9)(p14;q34.2) | CI14 | D6 | 4BB | Biopsy Cells: del(X)(q11.2q28)(~91.27Mb),del(2)(p14q37.3)(~178.40Mb),+9,-21 | 0 |
|  |  |  |  |  |  |
|  |  |  |  | BRD: Euploid |  |
|  |  |  |  |  |  |
| 45,XX,rob(14;21)(q10;q10) | CI15 | D5 | 4BB | Biopsy Cells: +15(~41%) | 0 |
|  |  |  |  |  |  |
|  |  |  |  | BRD: +X,dup(5)(p15.33p15.2)(~11.00Mb),dup(8)(p23.3p22)(~16.00Mb),dup(8)(p22p11.1)(~28.50Mb),dup(8)(q24.22q24.3)(~14.86Mb),+22 |  |
|  |  |  |  |  |  |
| 45,XX,rob(14;21)(q10;q10) | CI16 | D6 | 4BB | Biopsy Cells: del(15)(q11.2q13.1)(~6.00Mb) | 0 |
|  |  |  |  |  |  |
|  |  |  |  | BRD: -1,+2,-3(~45%),+8(~53%),+12(~62%),+14(~50%),-16(~45%),+17(~51%),+18,+20,+21,-22 |  |
|  |  |  |  |  |  |
| 46,XX,t(1;13)(q43;q12.2) | CI17 | D5 | 4BB | Biopsy Cells: Euploid | 0 |
|  |  |  |  |  |  |
|  |  |  |  | BRD: -3(~52%),dup(5)(p14.3q35.3)(~160.42Mb,~34%),dup(20)(p13q13.12)(~44.00Mb,~35%) |  |
|  |  |  |  |  |  |
| 46,XY,der(14;21)(q10;q10) | CI18 | D6 | 4BC | Biopsy Cells: Euploid | 0 |
|  |  |  |  |  |  |
|  |  |  |  | BRD: +1,+5(~67%),-6,-8(~66%),+10(~67%),-16(~46%),+17(~48%),+19,+20(~65%),-21(~40%),-22 |  |
|  |  |  |  |  |  |
| 46,XX,inv(8)(p12q12) | CI19 | D5 | 4BC | Biopsy Cells: dup(8)(p23.2p22)(~12.00Mb,~57%) | 0 |
|  |  |  |  |  |  |
|  |  |  |  | BRD: del(1)(p34.3q44)(~209.75Mb,~42%),+3(~41%),-4,-8(~53%),del(10)(p15.3q24.1)(~98.00Mb),+11,-12(~66%),+16(~65%),+18(~69%) |  |
|  |  |  |  |  |  |
| 46,XX,t(2;11)(q37;q21) | CI20 | D6 | 4CB | Biopsy Cells: Euploid | 0 |
|  |  |  |  |  |  |
|  |  |  |  | BRD: +15,+20(~59%) |  |
|  |  |  |  |  |  |
| 45,X,[29]/46XX[23] | CI21 | D6 | 4BB | Biopsy Cells: Euploid | 0 |
|  |  |  |  |  |  |
|  |  |  |  | BRD: dup(4)(q12q35.2)(~136.65Mb,~56%),dup(13)(q12.13q13.3)(~12.00Mb) |  |
|  |  |  |  |  |  |
| 46,XY,t(4;9)(q16;q13.3) | CI22 | D5 | 4BB | Biopsy Cells: Euploid | 0 |
|  |  |  |  |  |  |
|  |  |  |  | BRD: -3(~63%) |  |
|  |  |  |  |  |  |
| 46,XY,t(10;21)(p12;q22.1) | CI23 | D6 | 4BC | Biopsy Cells: Euploid | 0 |
|  |  |  |  |  |  |
|  |  |  |  | BRD: -9(~37%),dup(17)(p13.3q11.2)(~31.00Mb,~49%),-22(~48%) |  |
|  |  |  |  |  |  |
| 46,XX,t(1;3)(p34.1;q23) | CI24 | D6 | 4BC | Biopsy Cells: del(1)(p36.33p34.2)(~42.40Mb),dup(3)(q22.1q29)(~66.40Mb) | 0 |
|  |  |  |  |  |  |
|  |  |  |  | BRD: -8(~50%),+9(~42%),+13(~46%),+15(~44%),-16(~56%),+17(~65%),+22 |  |
|  |  |  |  |  |  |
| 46,XX,t(1;3)(p34.1;q23) | CI25 | D6 | 4BC | Biopsy Cells: dup(1)(p36.33p34.2)(~41.40Mb,~63%),dup(1)(p34.2q44)(~206.65Mb),del(3)(p26.3q22.1)(~131.80Mb) | 0 |
|  |  |  |  |  |  |
|  |  |  |  | BRD: Euploid |  |
|  |  |  |  |  |  |
| 45,XX,psu dic(18;15)(p11.32;p11.32) | CI26 | D6 | 6CB | Biopsy Cells: del(8)(p23.3p21.1)(~27.60Mb) | 0 |
|  |  |  |  |  |  |
|  |  |  |  | BRD: dup(8)(p23.3p21.1)(~28.50Mb) |  |
|  |  |  |  |  |  |
| 46,XX,t(3;12)(q23;q15) | CI27 | D6 | 4BB | Biopsy Cells: Euploid | 0 |
|  |  |  |  |  |  |
|  |  |  |  | BRD: -9(~52%) |  |
|  |  |  |  |  |  |
| 46,XX,inv(8)(q12;q24.2) | CI28 | D5 | 4BB | Biopsy Cells: Euploid | 0 |
|  |  |  |  |  |  |
|  |  |  |  | BRD: -10(~48%),-21(~47%) |  |
|  |  |  |  |  |  |
| 46,XX,inv(8)(q12;q24.2) | CI29 | D6 | 4BC | Biopsy Cells: del(2)(q21.1q37.3)(~112.00Mb),+16 | 0 |
|  |  |  |  |  |  |
|  |  |  |  | BRD: dup(20)(p13p11.23)(~21.00Mb,~59%) |  |
|  |  |  |  |  |  |
| 46,XX,inv(9)(p11q13) | NA1 | D5 | 4AA | Biopsy Cells: Euploid | — |
|  |  |  |  |  |  |
|  |  |  |  | BRD: NA |  |
|  |  |  |  |  |  |
| 46,XX,t(3;4)(q26.3;q27) | NA2 | D5 | 4BB | Biopsy Cells: Euploid | — |
|  |  |  |  |  |  |
|  |  |  |  | BRD: NA |  |
|  |  |  |  |  |  |
| 46,XY,t(2;16)(q36;q21) | NA3 | D6 | 5BB | Biopsy Cells: dup(2)(q35q37.3)(~22.20Mb),del(16)(q21q24.3)(~32.95Mb) | — |
|  |  |  |  |  |  |
|  |  |  |  | BRD: NA |  |
|  |  |  |  |  |  |
| 46,XY,t(9;17)(q22;p11.2) | NA4 | D6 | 4BB | Biopsy Cells: -X | — |
|  |  |  |  |  |  |
|  |  |  |  | BRD: NA |  |
|  |  |  |  |  |  |
| 46,XY,t(11;12)(q24.1;q22) | NA5 | D6 | 4BB | Biopsy Cells: +11,-22 | — |
|  |  |  |  |  |  |
|  |  |  |  | BRD: NA |  |
|  |  |  |  |  |  |
| 46,XX,t(4;20)(p10;p10) | NA6 | D6 | 4BC | Biopsy Cells: del(4)(p16.3q13.1)(~61.60Mb),del(9)(p24.3p13.1)(~39.00Mb),dup(9)(q21.11q34.3(~70.40Mb),-13,dup(20)(p11.23q13.33)(~44.43Mb) | — |
|  |  |  |  |  |  |
|  |  |  |  | BRD: NA |  |
|  |  |  |  |  |  |
| 46,XX,t(3;12)(q23;q15) | NA7 | D6 | 4BC | Biopsy Cells: Euploid | — |
|  |  |  |  |  |  |
|  |  |  |  | BRD: NA |  |
|  |  |  |  |  |  |
| 46,XY,t(1;4)(q25;q24.3) | NA8 | D6 | 4BC | Biopsy Cells: | — |
|  |  |  |  |  |  |
|  |  |  |  | BRD: NA |  |
|  |  |  |  |  |  |
| 45,XX,der(13;15)(q10;q10) | NA9 | D6 | 4BC | Biopsy Cells: +15 | — |
|  |  |  |  |  |  |
|  |  |  |  | BRD: NA |  |
|  |  |  |  |  |  |

**Note:** BRD: biopsy-related droplet; CNV: copy number variation; D5: Day 5; D6: Day 6; CC: complete concordance; PC: partial concordance; CI: complete inconsistency; NA: not available, indicating that no interpretable BRD CNV result was obtained. In the consistency column, 2 indicates complete concordance, 1 indicates partial concordance, and 0 indicates complete inconsistency between biopsy-cell and BRD CNV profiles. “—” indicates that consistency assessment was not applicable.
